# Prediction of complex human diseases from pathway-focused candidate markers by joint estimation of marker effects: case of chronic fatigue syndrome

**DOI:** 10.1186/s40246-015-0030-6

**Published:** 2015-06-11

**Authors:** Madhuchhanda Bhattacharjee, Mangalathu S. Rajeevan, Mikko J. Sillanpää

**Affiliations:** School of Mathematics and Statistics, University of Hyderabad, Hyderabad, 500046 India; Division of High-Consequence Pathogens & Pathology, Centers for Disease Control and Prevention, Atlanta, 30333 USA; Departments of Mathematical Sciences, Biocenter Oulu, University of Oulu, Oulu, FIN-90014 Finland

**Keywords:** Chronic fatigue syndrome, Phenotype prediction, Cross-validation, SNP, Bayesian analysis

## Abstract

**Background:**

The current practice of using only a few strongly associated genetic markers in regression models results in generally low power in prediction or accounting for heritability of complex human traits.

**Purpose:**

We illustrate here a Bayesian joint estimation of single nucleotide polymorphism (SNP) effects principle to improve prediction of phenotype status from pathway-focused sets of SNPs. Chronic fatigue syndrome (CFS), a complex disease of unknown etiology with no laboratory methods for diagnosis, was chosen to demonstrate the power of this Bayesian method. For CFS, such a genetic predictive model in combination with clinical evidence might lead to an earlier diagnosis than one based solely on clinical findings.

**Methods:**

One of our goals is to model disease status using Bayesian statistics which perform variable selection and parameter estimation simultaneously and which can induce the sparseness and smoothness of the SNP effects. Smoothness of the SNP effects is obtained by explicit modeling of the covariance structure of the SNP effects.

**Results:**

The Bayesian model achieved perfect goodness of fit when tested within the sampled data. Tenfold cross-validation resulted in 80 % accuracy, one of the best so far for CFS in comparison to previous prediction models. Model reduction aspects were investigated in a computationally feasible manner. Additionally, genetic variation estimates provided by the model identified specific genetic markers for their biological role in the disease pathophysiology.

**Conclusions:**

This proof-of-principle study provides a powerful approach combining Bayesian methods, SNPs representing multiple pathways and rigorous case ascertainment for accurate genetic risk prediction modeling of complex diseases like CFS and other chronic diseases.

**Electronic supplementary material:**

The online version of this article (doi:10.1186/s40246-015-0030-6) contains supplementary material, which is available to authorized users.

## Introduction

Human genetic studies generally focus on identifying genes associated with diseases and subsequently using the significant genetic information for phenotypic predictions. Statistical methods vary with each of these goals, with methods for gene identification becoming increasingly effective with genome-wide association studies (GWAS). However, modeling principles for phenotypic predictions of complex human diseases remain largely unexplored even in this era of GWAS [1, 2]. Phenotypic predictions in human data sets have been modeled using regression models with a few single nucleotide polymorphisms (SNPs) having strong association identified by GWAS, but this practice of using only a few genetic markers has been disappointing due to its low predictive power [3]. A similar problem known as “the problem of missing heritability” is encountered when only highly associated markers are used to estimate heritability of complex traits [4, 5]. However, as demonstrated recently with human height and complex diseases, the missing heritability problem can be alleviated when linear regression models include information from all markers in the GWAS [6–8]. Such models can be seen as finite locus approximations for polygenic models, being representative for linkage disequilibrium (LD) structure in population, and they should therefore provide useful predictions even in the presence of a large number of rare variants.

Bayesian variable selection [9, 10] and the frequentist regularization methods [11, 12] have gained attention to perform parameter estimation and variable selection simultaneously in phenotype-marker association analysis. These methods generally perform well in selecting trait-associated loci or loci in LD to estimate genomic breeding values in animals and plants [13, 14]. Lee et al. [15] considered that methods for predictions of phenotypes and genomic breeding values may employ similar tasks and can be successfully substituted for one another. Generally, it is known that the marker density (the length of the genome), population LD, and number of individuals in the learning sample will all have a strong influence on the prediction accuracy [1]. Moreover, simplicity of the genetic architecture and heritability of the trait are also the key factors for the prediction success. Animal and plant breeding literature includes numerous simulation studies on how these factors influence prediction accuracy, see, e.g., references in [1]. Generally, from studies of different prediction methods applied to plant and animal data sets, one can conclude that variable selection approaches seem to work most efficiently for oligogenic traits with sparse genetic architectures in the presence of moderate LD among markers. On the other hand, approaches based on mixed models and marker-estimated covariance structures [1, 16] seem to work well for polygenic traits or oligogenic traits in the presence of strong LD among markers (which will make oligogenic data sets to have large numbers of trait-associated markers like polygenic traits). Certain modern classification methods [17] and Bayesian subset selection methods [18, 19] have been applied to predict chronic disease or to find important subsets of SNPs contributing to chronic diseases with small data sets. In general, combining multiple sources of information may lead to more accurate phenotype prediction [12, 20, 21]. This approach has been confirmed with simulations but its benefit with real data is still questionable, especially with complex phenotypes [19]. We consider here information from a carefully selected set of pathway-focused SNPs and applied Bayesian methods in the context of association mapping to model phenotype status. In association studies with SNPs, it is difficult to distinguish the most associated SNPs in regions of high LD. In such situations, it may be informative to inspect for stable association signals after first smoothing the signals in regions of high LD [22]. On this basis, we hypothesize that more stable and accurate phenotypic prediction may be achieved after smoothing pathway-focused SNP effects in regions of high LD. Our goals here are to provide genomic prediction of an individual’s disease status as a sum of SNP effects, which depend on individual’s genotype pattern at SNPs. This is done by means of model-averaged estimation of genetic effects used to weight individual SNPs. For this, we use Bayesian methods in genetic association studies and model-averaged estimate of genetic effects that we call as weighted genetic variation (WGV) [23]. Our Bayesian methods can perform variable selection and parameter estimation simultaneously and can induce the sparseness and smoothness of the estimated SNP effects [19, 23]. Smoothness of the SNP effects is obtained by the explicit modeling of the covariance structure of the SNP effects. We evaluated our Bayesian modeling principle and methods for modeling of chronic fatigue syndrome (CFS), as an example of a complex disease where its diagnosis remains elusive with the need for improved analytical approaches for gene identification and accurate phenotypic prediction [24].

## Methods

### Subjects and illness classification

This study adhered to human experimental guidelines of US Department of Health and Human Services and the Helsinki Declaration. The Centers for Disease Control Human Subjects committee approved the study protocol, and all subjects gave written informed consent. Subject recruitment, clinical evaluation, laboratory tests, and their classification were described previously [25]. Briefly, 227 subjects were recruited from Wichita, KS, USA, as part of a 2-day in-hospital evaluation of unexplained fatigue. These subjects were identified from a surveillance cohort of 7162 fatigued and non-fatigued subjects who were originally screened from 56,146 adult residents, 18 to 69 years of age. During the 2-day hospital stay, symptoms and exclusionary medical and psychiatric conditions were reevaluated for all 227 subjects. Following the 2-day hospital study, all subjects were classified based on all aspects specified in 1994 CFS case definition [26] and Medical Outcomes Short-Form, Multidimensional Fatigue Inventory and Symptom Inventory cutoff scores to include measures on the functional impairment, fatigue, and accompanying symptom complex that characterize CFS. Following this classification, 124 subjects were excluded because of medical or psychiatric exclusionary conditions or insufficient criteria to classify as CFS. Of the 103 remaining subjects, 101 subjects with genotype data were classified as CFS (43 subjects) and non-fatigued (NF; 58 subjects) healthy controls. The demographic characteristics along with the type of disease onset (gradual vs. sudden) of subjects in this study are given in Table 1.

**Table 1:** Demographic and other characteristics of the subjects selected for analysis

Factor	Categories	NF subjects (*n* = 58)	CFS subjects (*n* = 43)
Age (years)	MQMQM^a^	31.0/44.3/51.5/56.0/69.0	27.0/46.5/51.0/57.5/69.0
Sex (*n*)	Female/male	46/12	36/7
Race (*n*)	White/Black/others	54/2/2	40/1/2
BMI	MQMQM	16.0/25.3/29.0/32.0/40.0	23.0/26.0/29.0/32.5/40.0
Onset^b^	Gradual/sudden	14/1	36/6

^a^MQMQM represents the minimum, first quartile, median, third quartile, and maximum, respectively

^b^Onset represents gradual vs. sudden onset of illness. This information is available for all but one CFS subject. For NF subjects, onset information is relevant to only 15 individuals with past report of chronic fatigue

### SNP selection, genotyping, and annotation

Because of the reported associations of CFS with perturbations in hypothalamic-pituitary-adrenal (HPA) axis and immune functions, we selected a total of 39 candidate genes implicated with the central nervous system (CNS) (30 genes) or immune and inflammation functions (nine genes) to determine the accuracy CFS prediction based on combinations of SNPs (Additional file 1: Table S1). There were a total of 167 SNPs in all candidate genes (137 SNPs in genes implicated with CNS and 30 SNPs in genes implicated with immune and inflammation). There were a total of 23 SNPs in X-chromosomes, and these were in two genes of serotonergic neurotransmission (*HTR2C* and *MAOA*). SNPs were selected from the SNP database (dbSNP) of National Center for Biotechnology Information database, Applied Biosystem’s SNPBrowser™ or from the literature. All SNP markers had a minor allele frequency ≥10 % with the exception of *HTR2A* rs6314 (6.2 %), a non-synonymous SNP. DNA extraction and genotyping were done as described earlier [27]. Most SNPs (158 out of 167) were genotyped using validated TaqMan genotyping assay kits (Applied Biosystems, CA, USA) and the 7900 Sequence detection system (Applied Biosystems). Eight SNPs were genotyped by pyrosequencing. Genotyping for one polymorphism in *SLC6A4* designated 5-HTTLPR was conducted using gel-based assays [28]. SNP annotation was done using SPOT algorithm as implemented in the web-based tool accessible at https://spot.cgsmd.isi.edu/submit.php [29]. It may be noted that SNP selection was done in the design stage of this study before genotyping while in some other studies, statistical prescreening procedures such as sure independent screening were applied after genotyping to reduce the dimensionality [30, 31].

### Model

We applied Bayesian logistic association models [18, 19] using subset of SNPs to predict disease status (*y*_*i*_) of the individual *i*. Our hierarchical model structure underlying our predictive model is almost identical to that presented earlier by us [23] for LD mapping.

The logistic association model for SNP data (of individual *i*) can be written as:

$$ \mathrm{Logit}\left({p}_i\right)=\alpha +{\displaystyle \sum_{l\in {M}_A}\left({\beta}_{l,1}\left(2-{m}_{i,l}\right)+{\beta}_{l,2}{m}_{i,l}\right){I}_l}+{\displaystyle \sum_{l\in {M}_S}\frac{1}{2}\;\left({\beta}_{l,1}\left(2-{m}_{i,l}\right)+{\beta}_{l,2}{m}_{i,l}\right){I}_l}. $$

Here, the logistic link function, $\mathrm{Logit}\left({p}_i\right)= \ln \left[\frac{p_i}{1-{p}_i}\right]= \ln \left[\frac{P\left({y}_i=1\Big|m\right)}{1-P\left({y}_i=1\Big|m\right)}\right]$, *α* is an intercept, (*β*_*l*,1_ and *β*_*l*,2_) are genetic effects of SNP *l. I*_*l*_’s are the indicator variables taking care of variable selection to select subset of SNPs to the model (see below for more details). The SNPs have been reorganized into two groups according to whether they are on sex chromosomes (i.e., *l* ∊ *M*_*S*_) or autosomal chromosomes (i.e., *l* ∊ *M*_*A*_). The genotype value *m*_*i*,*l*_ of individual *i* at SNP *l* is represented numerically by 0 for homozygote AA, 1 for heterozygote AB, and 2 for homozygote BB. Our model for the genetic effects assumes an additive model, where an effect of the heterozygote (*β*_*l*,1_ + *β*_*l*,2_) is in the middle of the effects of two homozygotes (*β*_*l*,1_ + *β*_*l*,1_ and *β*_*l*,2_ + *β*_*l*,2_). It is over-parameterized to improve the mixing and convergence properties of the Markov chain Monte Carlo (MCMC) sampling algorithm to yield better parameter estimates. The usual practice would be to set the first genetic effect to zero and have one parameter only for the other genetic effect. The absolute difference of the two effects here reflects the effect size of the SNP. Note that the factor of one half is introduced for the SNPs on the sex chromosome due to female X-chromosome mosaicism and the adjustment by half can be used for male only after assuming all SNPs on their X-chromosome are homozygous (cf. [32]).

We assume a priori that there is only a small subset of important SNPs that are useful to predict disease status. In these predictive models, subset selection of important SNP effects to the predictive model is based on the use of indicator variables (*I*_*l*_, *l* = 1,…, *M*), all of which either equals one (inclusion) or zero (exclusion) depending on the importance of particular SNP (see, e.g., [9]). Here, *M* is a number of SNPs. However, in reality for closely situated SNPs, it is difficult to distinguish the effects of individual SNPs due to LD. Thus, LD pattern among SNPs in the linked region is explicitly modeled as dependency prior for variable selection indicators in our model as described below and reported earlier in [23].

#### Model for missing SNP data

Missing SNP data are handled in a similar manner as any other model parameter. Thus, prior distributions are assigned to all missing SNP data. We assume a priori that the missing SNP genotypes occur at random and independently within and across SNPs (in the sense that the probability that the genotype is missing is not dependent on the true genotype pattern at the locus or at any of its neighboring loci). The prior distribution *p*(*m*_*i*,*l*_) of a missing genotype *m*_*i*,*l*_ under the Hardy-Weinberg equilibrium is a binomial distribution, where both alleles have equal occurrence probabilities within the locus (i.e., *p*(*m*_*i*,*l*_) = 0.25, 0.5, and 0.25 for three genotypes AA, AB, and BB, respectively). Additionally, an independence over loci (linkage equilibrium) is assumed for missing SNP genotypes as $p(m)={\displaystyle \prod_{i=1}^Np\left({m}_{i,1},\dots, {m}_{i,M}\right)={\displaystyle \prod_{i=1}^N{\displaystyle \prod_{l=1}^Mp\left({m}_{i,l}\right)}}}$. During MCMC sampling, our phenotypic model (likelihood) gives information on which genotype value is most likely in the light of the data. Thus, we let the data to speak in our missing data model. For meta-analysis purposes, where SNP genotypes at some data sets may be missing for all the individuals, one may want to consider to predict the SNP genotypes with reasonable accuracy using genotype imputation methods (utilizing LD correlation structure between neighboring SNPs) based on HapMap and 1000 genomes project reference panels [33, 34].

#### Priors for α*,* β*,* I

Prior specification is intrinsically subjective, and specifying prior that will satisfy everyone and/or every aspect might be unachievable. We adopt the method where priors reflect our intuitive knowledge but are also useful in avoiding some potential pitfalls and help reduce the computational burden. The parameter *α* relates to the intercept term of the regression which in this case with the Logit function would be close to zero (with 43 CFS cases and 58 NF controls). In the case of regression modeling a quantitative phenotype, the variance of the intercept parameter can be related to the scale of the phenotype. Here, we have binary phenotype; thus, without further information on the variability, we assume a standard normal distribution for the intercept parameter *α*.

The parameters *β* and *I* together determine which SNPs potentially have effect on the phenotype and the extent of this effect.

Following [23], we denote the vector of (genetic or physical) distances between the SNPs with *d* = *(d*_*2*_,*…*, *d*_*M*_*).* We also use a smoothing parameter *λ* for neighboring SNPs which allows us to model dependence of two adjacent SNPs. Our prior probability for each SNP to be involved in the model is $\left.P\Big({I}_l=1\right|s\Big)=s=\frac{1}{M}$, which corresponds to assuming only a single marker to be important predictor in the model (see [23] for details). However, SNPs exhibiting strong LD in a single genomic region would change these probabilities, and we model this by a Markov model where the extent of LD is decaying according to the distance information [23]. This is, $P\left({I}_1,\dots, {I}_N\left|s\right.,\lambda, d\right)=P\left({I}_1\left|s\right.\right){\displaystyle \prod_{l=2}^MP\left({I}_l\right|}{I}_{l-1},s,\lambda, {d}_l\Big)$. Given the state of inclusion indicators at locus (*l −* 1), the transition matrix for the inclusion indicators for locus *l* is given by

$$ \left(\begin{array}{cc}\hfill {e}^{-\lambda {d}_l}+\left(1-{e}^{-\lambda {d}_l}\right)\left(1-s\right)\hfill & \hfill \left(1-{e}^{-\lambda {d}_l}\right)s\hfill \\ {}\hfill \left(1-{e}^{-\lambda {d}_l}\right)\left(1-s\right)\hfill & \hfill {e}^{-\lambda {d}_l}+\left(1-{e}^{-\lambda {d}_l}\right)s\hfill \end{array}\right). $$

Genetic effects (*β*_*l*,1_ and *β*_*l*,2_) of each locus are assigned marginally a priori using a scale-mixture representation of Student’s *t* distribution (e.g., [35]). This means that genetic effects at each locus are first assumed to be normally distributed with common variance *σ*_*l*_^2^ which again is assumed to be a priori inverse gamma distributed. In this model, a vector of locus-specific genetic variance components *σ*^2^ = (*σ*_1_^2^,…, *σ*_*M*_^2^), over the *M* loci, controls the corresponding genetic effect parameters under a normal model. We further make the following conditional independence assumption that given *σ*^2^ and *s*, the locus indicators *I* and genetic effects *β* are independent. Thus, prior distribution *P*(*β*_*l*,*k*_|*σ*_*l*_^2^) for genetic effects *β*_*l,k*_(*k* = 1,2) was assumed to be normal *N*(0, *σ*_*l*_^2^) with locus-specific variance component *σ*_*l*_^2^.

#### Priors for hyper-parameters σ_l_^2^, λ

The prior for genetic variance *P*(*σ*_*l*_^2^) at locus *l* was given an inverse *gamma* (1, 1), and consequently, *P*(*σ*^2^) = *Π*_*l*_^*M*^_= 1_*P*(*σ*_*l*_^2^). The smoothing parameter *λ* is given a wide prior of *gamma* (1, 0.01) which has both the mean and standard deviation as 100. This parameter helps to eliminate spurious associations but strengthens the real association signals. However, since we utilize a common smoothing parameter over multiple locations of the genome, it lacks intuitive explanation compared to situation where it is used for a single densely mapped genomic region. There, this parameter can be thought to roughly represent the time since the relevant mutation affecting the phenotype (see [23]).

#### Complete model

The relevant joint density to derive the posterior of the parameters of interest is then obtained using the following expression which is based on the above and utilizes appropriate conditional independence assumptions:

$$ \begin{array}{l}p\left(y,I,\alpha, \beta, {\sigma}^2,\lambda, m\Big|s,d\right)\kern0.5em =\kern0.5em p\left(y\Big|m,I,\alpha, \beta \right)\hfill \\ {}\kern10.3em \times \kern0.5em p\left(I\Big|\lambda, s,d\right)p\left(\lambda \right)\hfill \\ {}\times \kern0.5em p\left(\alpha \right)p\left(\beta \Big|{\sigma}^2\right)p\left({\sigma}^2\right)\hfill \\ {}\times \kern0.5em p(m)\hfill \end{array} $$

### Weighted genetic variation and heuristic model reduction

The weighted genetic variation (WGV_l_ = |*β*_*l,*1_ *− β*_*l,*2_|*I*_*l*_) at locus *l*, (*l = 1,…, M*), is computed as a product of absolute difference of the genetic effects |*β*_*l,*1_ *− β*_*l,*2_| and the inclusion indicator (*I*_*l*_).

A heuristic model reduction method was used to speed up the estimation by determining the number of SNP predictors that can be reduced without loss of predictive ability. Using the percentiles of WGV (Additional file 1: Table S2) as cutoff/threshold, a selected set of SNPs was retained in the model with two critical components derived from the overall model as follows. For the indicators, the joint posterior distribution of the indicators from the full model was used where the outcome of the spike-n-slab technique over the MCMC simulation for full model was stored and reused for model reduction purposes. For the remaining SNPs (with WGV lower than the threshold value), the individual-level genotype information were not used, and thus, these SNPs would effectively cease to act as covariates in the model (see Additional file 1 for detailed information on WGV and heuristic model reduction).

### *K*-fold cross-validation

Cross-validation methods [36] give a better assessment of model predictive performance for new data, i.e., phenotypic predictions of individuals whose phenotypes and genotypes have not been involved in the learning sample. The hold-out or split-sample method, in which the data is split into training and testing sets, is the simplest kind of cross-validation. While this method assesses model performance on real prediction situation with new data, it is subjective to the choice of the partition of the data into training and testing sets. *K*-fold cross-validation is one way to improve over the split-sample method. The data set is divided into *K* (approximately) equal subsets, and the hold-out method is repeated *K* times, by which every data point gets to be in a test set exactly once and gets to be in a training set *K* − 1 times. We have used *K* = 10 which is also one of the most popular choices of *K* [37].

One major disadvantage of *K*-fold cross-validation is that the time taken would also be typically *K* times that required for estimation based on whole data. Using data on all 167 SNPs, a tenfold cross-validation would require approximately 150 min per MCMC iteration. We retained information from all SNPs since CFS phenotype is complex and there could be loss of predictive ability with reduced number of SNPs. Therefore, instead of reducing the number of SNPs in the model to reduce the time, we made a few modifications to the full model (“Model” section) as noted in the supplementary file.

### Model implementation

The models were implemented in WinBUGS software [38], a special software to carry out MCMC simulation from posterior of complex models. The simulations were started with random initial values and were run for several thousands of iterations. Convergence of MCMC chains was monitored by visual inspection of MCMC trace plots with respect to several different model parameters and also by assuring that low values of the MCMC error have been reached for all critical model parameters. Software codes are available in the supplementary file and at http://www.rni.helsinki.fi/~mjs/.

### Comparison with competing prediction methods

To compare our method, certain predictions were made for the same data set with generalized linear model versions of LASSO and ridge regression [11, 12] using R-package “penalized” [39]. LASSO model assumes independence among predictors while ridge regression can handle collinearity among them. We also compared our Bayesian model assuming independence between predictors (i.e., prior independence among indicator variables). This can provide information on the importance of the dependence structure to the model. LASSO and ridge regression analyses were performed using the same over-parameterized model as in the Bayesian analysis which had own coefficient for each allele. Before LASSO and ridge regression analyses, we imputed missing data (once) using the Bayesian missing data model. However, this resulted in rank-deficient data matrix in which we eliminated 15 markers to make matrix acceptable for the R-package. To find the best tuning parameter value in LASSO (and ridge regression), we tried 55 (and 94) different values in range [0.1, 1000] (and [0.01, 25000]), respectively. In the Bayesian analysis, the priors and other settings were kept the same as earlier.

## Results

### CFS in-data prediction using full model

In-data prediction basically uses the training data itself to model the phenotype outcome. The data described earlier in the “Subjects and illness classification” and “SNP selection, genotyping, and annotation” sections and the model developed by us (presented in the “Model” section) were used to evaluate the goodness of fit of the model to predict CFS for 101 subjects (with 43 CFS and 58 NF individuals) using all the SNP data. This in-data prediction model, among other things, provided estimates of WGV of the SNPs, which are combined measures of their selection probability in the model and degree of effect on the phenotype (Additional file 1: Table S2). This is computed as a random variable based on the product of the indicator and absolute difference of the genetic effects for a locus, i.e.

WGV_l_ *= |β*_*l,*1_ *− β*_*l,*2_*|I*_*l*_ for the *l*-th locus, *l = 1,…, M*. The MCMC sampling from the posterior also enables us to estimate the WGVs. SNPs rs2288831 (*IL12B*), rs2071376 (*IL1A*), rs2069718 (*IFNG*), rs846906 (*HSD11B1*), rs1923884 (*HTR2A*), rs1799836 (*MAOA*), and rs1396862 (*CRHR1*) were among the top 10 SNPs with the highest genetic effect on the phenotype as measured by WGV (Table 2). For this particular CFS data using all 167 SNPs, this full model showed 100 % goodness of fit, probably due to over-fitting in a small data set. This necessitates extensive cross-validation of the model with unseen test data.

**Table 2:** Top 10 genetic markers associated with CFS based on weighted genetic variation (WGV) estimated by the Bayesian model

SNP ID	Proxy SNP	Gene symbol^a^	SNP annotation^a^	WGV	SE of WGV^b^
rs2288831	rs3212227	*IL12B*	Intron (UTR-3)	3.95	0.0299
rs2071376		*IL1A*	intron	3.6	0.0296
rs2069718		*IFNG*	intron	3.34	0.0272
rs846906		*HSD11B1*	intron	3.29	0.0337
rs1923884		*HTR2A*	intron	3.16	0.0324
rs1799836		*MAOB*	Intron	2.56	0.0394
rs363236	rs3814230	*SLC18A2 (PDZD8)*	UTR-3 (synonymous codon)	2.31	0.0272
rs1396862	rs1218523	*CRHR1 (IMP5)*	Intron (missense codon)	2.31	0.0334
rs891512	rs743507	*NOS3*	Intron	2.18	0.0287
rs1124492	rs46220755	*DRD2*	Intron	2.02	0.0312

^a^Gene symbol and SNP annotation in parenthesis correspond to proxy SNPs, if different from the genotyped SNPs for the model

^b^SE of WGV standard error of weighted genetic variation

### Impact of CFS prediction using variable number of SNPs

**Fig. 1:**
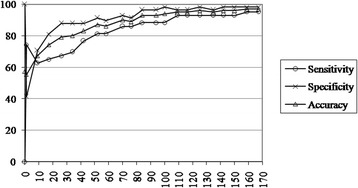
Impact of varying the number of SNPs on prediction performance, as measured by sensitivity, specificity, and accuracy

We examined if the number of SNP predictors can be reduced without loss of predictive ability by implementing a heuristic model reduction. Using percentiles from the estimated WGV, an increasing number of SNPs were included in the model with appropriate parameters, while the effect of the rest was adjusted as described above. As expected, with the increased number of SNPs, there was a corresponding increase in the model prediction accuracy (see Fig. 1 and Table 3). Although prediction accuracy reached near perfection (accuracy 97 %) with nearly all SNPs (159 out of 167), accuracy remained still close to perfection (accuracy 95 %) using a combination of close to 100 SNPs (35th percentile of WGV). Accuracy remained high (90 %) even with the top 70 SNPs in WGV. Accuracy decreased with fewer SNPs in the model, although we also obtained 79 % accuracy with combinations of 26 SNPs ranked top in WGV.

**Table 3:** Increase in accuracy with increasing number of SNPs in the predictive model with individual-level allelic information

Percentile^a^	Cutoff for weighted genetic variation	No. of SNPs	Sensitivity	Specificity	Accuracy
100	3.95	1	74.42	41.38	0.55
95	2.13	9	62.79	70.69	0.67
90	1.30	17	65.12	81.03	0.74
85	1.07	26	67.44	87.93	0.79
80	0.96	34	69.77	87.93	0.80
75	0.82	42	76.74	87.93	0.83
70	0.79	52	81.40	91.38	0.87
65	0.74	58	81.40	89.66	0.86
60	0.70	70	86.05	93.10	0.90
55	0.68	76	86.05	91.38	0.89
50	0.63	84	88.37	96.55	0.93
45	0.58	94	88.37	96.55	0.93
40	0.54	100	88.37	98.28	0.94
35	0.51	109	93.02	96.55	0.95
30	0.48	116	93.02	96.55	0.95
25	0.45	125	93.02	98.28	0.96
20	0.42	135	93.02	96.55	0.95
15	0.41	142	93.02	98.28	0.96
10	0.36	150	93.02	98.28	0.96
5	0.32	159	95.35	98.28	0.97
0	0.00	167	100	100	1.00

^a^Percentiles are those for the estimated weighted genetic variation (WGV) under the full model

**Fig. 2:**
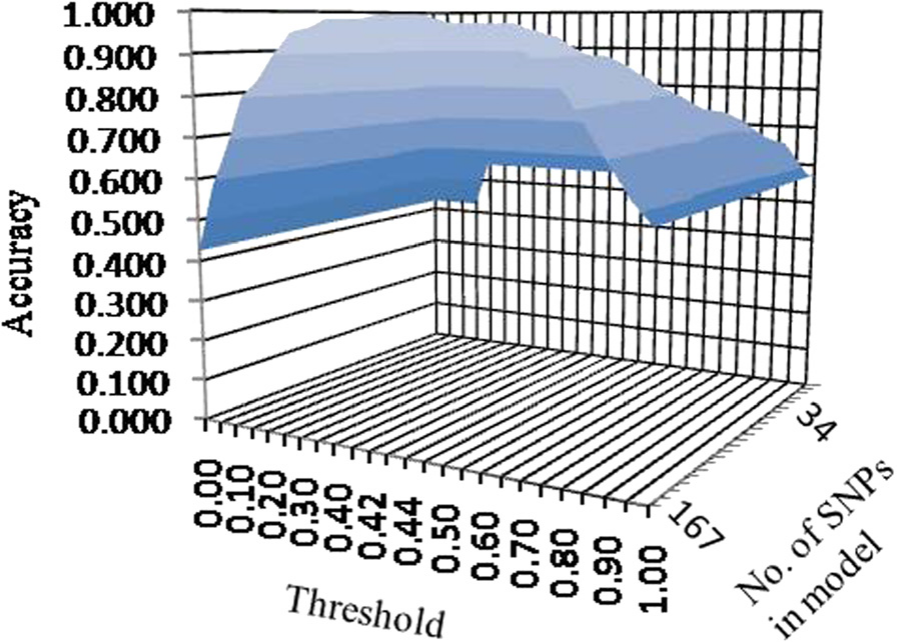
Impact of varying the threshold and the number of SNPs on prediction performance as assessed by the in-data prediction accuracy

The above utilized the cutoff probability of 0.5 to declare a case as CFS or otherwise. However, this threshold could also be varied. Figure 2 presents the predictive performance as measured by accuracy of prediction while varying the threshold probability and number of (unadjusted) SNPs in the model. As stated earlier, SNPs were considered in the increasing order of their WGV for inclusion/exclusion in the model. Thus, least effective SNPs are removed from the model at first. This illustrates that model accuracy is not highly susceptible to the choice of cutoff and is capable of staying at high level in an interval around the chosen 0.5 used for this analysis.

### Tenfold cross-validation of CFS prediction

**Fig. 3:**
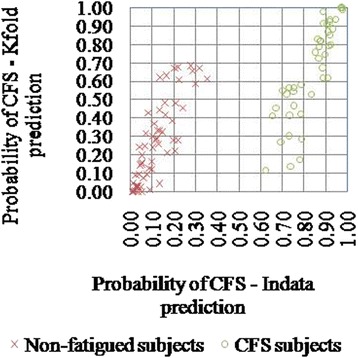
Probability of CFS at individual level as estimated by the *K*-fold cross-validation model and in-data prediction model

The cross-validation results on prediction are summarized using the standard sensitivity, specificity, false detection rate (FDR), and overall accuracy (Table 4). Computation time per iteration is approximately 50 min. As can be seen, the overall accuracy is still high (79 %) for the tenfold cross-validation. The overall sensitivity and specificity of the tenfold cross-validation were 74.4 and 82.8 %, respectively. Accuracy for individual sets varied between 70 and 100 %. At an individual level, the prediction probabilities were compared for the *K*-fold model and in-data model, and the results are presented as Fig. 3. We observe that those predicted with high probability/accuracy when the data is seen can still be recovered when the respective data is withheld. However, as this accuracy comes down, probability of correct prediction also comes down when the corresponding data is unseen. It should also be noted that the grouping of subjects based on overall cross-validation probability (>0.5) still predicts 14-fold higher risk for a subject to be classified as CFS (OR = 13.963; 95 % confidence interval 5.313–36.69; *p* value = 6.64 × 10^−9^).

**Table 4:** CFS prediction from in-data and tenfold cross-validations

Model and prediction type	Accuracy	Sensitivity	Specificity	FDR
In-data-unconstrained model	100.0	100.0	100.0	0.0
In-data-constrained model	100.0	100.0	100.0	0.0
*K*-fold-constrained model	79.2	74.4	82.8	23.8
*K*-fold-set1	72.7	60.0	83.3	25.0
*K*-fold-set2	70.0	75.0	66.7	40.0
*K*-fold-set3	80.0	75.0	83.3	25.0
*K*-fold-set4	100.0	100.0	100.0	0.0
*K*-fold-set5	70.0	50.0	83.3	33.3
*K*-fold-set6	70.0	50.0	83.3	33.3
*K*-fold-set7	70.0	75.0	66.7	40.0
*K*-fold-set8	70.0	75.0	66.7	40.0
*K*-fold-set9	100.0	100.0	100.0	0.0
*K*-fold-set10	90.0	80.0	100.0	0.0

For comparison, the same cross-validation analysis was done for LASSO, ridge regression, and Bayesian model without dependence (Table 5). The Bayesian approach (with and without dependence) clearly outperformed LASSO and ridge regression in accuracy and in sensitivity, but specificity was slightly better in penalized regression approaches. Including between SNP dependence to the Bayesian model improved sensitivity from 65.1 to 74.4. Otherwise, accuracy and specificity remained almost at the same levels for the two Bayes models.

**Table 5:** CFS prediction from tenfold cross-validation for competing methods

Model	Accuracy	Sensitivity	Specificity
Bayes with dependence	79.2	74.4	82.8
Bayes with independence	77.2	65.1	86.2
LASSO	59.4	20.9	87.9
Ridge regression	60.4	18.6	91.4

There were a total of 21 false predictions out of 101 predictions in this study. We investigated the source of these false predictions in terms of the sudden vs. gradual onset of chronic fatigue as reported by the subjects in the study. It appears that tenfold cross-validation is accurate for all individuals (six out of six) with sudden onset of CFS and one CFS subject with no onset information. All the erroneous predictions for CFS cases occurred only for those CFS cases with gradual onset (11 out of 36 CFS subjects with gradual onset). Among NF controls, 15 subjects reported previously being fatigued (14 gradual and 1 sudden onset) but did not meet the criteria for CFS, and three of these NF controls were predicted incorrectly. The remaining seven false predictions belonged to the 43 NF subjects who reported that they were never chronically fatigued before. These erroneous predictions potentially reflect heterogeneity in both cases and control subjects in the study.

## Discussion

This is a proof-of-principle study that presents a powerful genetic approach that can simultaneously rank SNPs based on their genetic effect and for prediction of complex phenotype based on a Bayesian logistic mixture modeling principle combined with biologically meaningful pathway-focused genetic markers and rigorous case ascertainment. We used CFS SNP data as an example to apply our analytical approach and to compare our model performance with LASSO and ridge regression as well as with results in the literature using other approaches. Our Bayesian prediction model is suitable for any data set that has genotype calls and subjects ascertained into binary phenotype, regardless of whether the illness is specialized or not.

Our modeling approach yielded 80 % accuracy after tenfold cross-validation with CFS data. This prediction accuracy is one of the best so far for this complex disease compared to other prediction models using the same data set. In comparison to other analytical methods, several modeling principles we used may have contributed to this high prediction accuracy. For example, we incorporated covariance due to LD between SNPs so that SNP effects of the predictive model depended on each other and dependence vanishes according to exponential decay with the genetic or physical distance [40]. Originally, closely after the single locus model of Conti and Witte [40], we presented our multi-locus model for genetic association mapping in small chromosomal segments [23]. After that, Malo et al. [41] and Tsai et al. [42] have expressed their own approaches to model covariance between SNP effects in genetic association analyses. Very recently, Fridley and Jenkins [43] also introduced an approach, which is closely related to our earlier model [23] for genetic association studies, see also [35, 44, 45] for the other alternatives.

A second methodological enhancement in our model is that it incorporates both sparse selection of trait-associated SNPs and smoothing of estimated SNP effects, and that this enhancement potentially provided competitive and accurate phenotype predictions in comparison to previous methods for CFS prediction. There are two previous reports [17, 46] on testing combinations of SNPs for prediction of CFS using the same subjects but a smaller set of (42 SNPs out of 167) SNPs used in this study. Because of the smaller set of SNPs, Goertzel et al. [46] used an enumerative approach with cross-validation by permutation and reported 76.3 % accuracy in predicting CFS. Huang et al. [17] compared three sets of classification methods (naïve Bayes model, support vector machine, and C4.5 decision tree algorithm) with 42 SNPs and found the naïve Bayes model with the wrapper-based feature selection to give the best overall sensitivity (64 %) and specificity (52 %). This comparison of our model having dependence structure with Huang et al. that does not have dependence structure [17] shows that dependence model like ours provides clear predictive advantage. Our study implies that models with dependence structure should be utilized more in methods addressing the prediction problem. While there are methods that take into account of dependence between SNPs for association mapping [46, 47], to the best of our knowledge, our Bayesian model is the only one that probabilistically models dependence between SNPs for prediction of binary phenotype. Moreover, since there are explicit differences in the models for association mapping and phenotypic prediction, methods developed for association mapping [46, 47] are not necessarily suitable or preferable for phenotypic prediction and vice versa.

Our heuristic model reduction method provided insight into the strength of Bayesian multi-locus association involving 100’s of SNPs in comparison to current regression models using a few genetic variants. In this analysis, prediction accuracy reached near perfection (accuracy 97 %) with nearly all SNPs (159 out of 167) and accuracy remained still close to perfection (accuracy 95 %) using a combination of close to 100 SNPs (35th percentile of WGV). Accuracy remained high (90 %) even with the top 70 SNPs in WGV but it decreased with fewer SNPs in the model, although we also obtained 79 % accuracy with 27 SNPs. These results clearly show that the higher the genetic information in the SNP profile, the greater will be the accuracy in prediction of complex diseases. Since the current computational approaches limit multi-locus analysis to only a few genetic variants, each with weak associations, current attempts to predict the individual genetic risk of complex disease traits have not been highly accurate, and their value/relevance was also questioned [48]. However, our results suggest that one can actually achieve reasonable predictive accuracy by using Bayesian predictions based on multi-locus association models involving 100’s of SNPs in a small set of genes. Prediction can be further improved with greater accuracy, sensitivity, and specificity, if the model includes genetic variation in hundreds of genes representing expanded set of multiple pathways implicated in immune, inflammation [49], and CNS functions [50]. With additional improvements in genetic variation data, modeling, and sample size, one can also conceive the potential of our approach to generate genetic signatures to delineate heterogeneity/subtypes in complex disease.

Other statistical considerations that favored high accuracy include model selection and estimation (including phenotype predictions) being done simultaneously. By this, we obtained estimates for posterior probability (uncertainty) of disease status and the degree of belief estimate for predicted disease phenotypes. These predictions, which are based on the Bayesian model averaging approach providing robust predictions [51], are however not based on a single best model but rather several different models where prediction of each model is weighted according to the corresponding posterior model probability. Model reduction aspects were investigated in a computationally feasible manner for an otherwise analytically intractable problem. We also showed accurate predictions from partial genotype data. That is, while the learning set contains information on complete set of SNPs, accuracy does not immediately drop if phenotype predictions are made for individuals which have genotypes measured from only a partial set of SNPs. We used indicator variables in the predictive models because they will make it possible to shrink coefficients at some unimportant positions exactly to zero. The same numerical property is difficult to obtain by tuning the value of the shrinkage parameter (which controls amount of shrinkage) in regularization models like the Bayesian LASSO [13], which do not contain indicators in the model. In those models, which have a single shrinkage parameter only, there is always trade-off between obtaining enough shrinkage for unimportant contributors and still maintaining good estimation ability for important positions (without having too much shrinkage).

It was reported recently that in order to achieve accurate prediction of human disease for unrelated individuals, it would take approximately a sample size of 350,000 [52]. This is because accuracy is dependent on the average relatedness between individuals in the population (i.e., effective population size structuring the LD) and genetic architecture of the trait. It was further noted that it can be accurate on small samples only for diseases that are determined by a limited number of genes which is unlikely to be true for a complex disease. It is thus possible that population ascertainment process that involved rigorous clinical evaluation of both cases and control subjects in this study might be an important factor, along with the strength of modeling and SNP selection, for prediction accuracy in this particular case. We are also aware of the rule of thumb that the number of independent predictors should not be greater than the one tenth of the number of samples in the smaller of the two outcome categories in logistic regression modeling. This limitation may be overcome by using the conventional two-stage procedure wherein significant markers are first evaluated from independent analysis, and then, the short-listed markers tested for prediction accuracy. Our analytical approach is to move away from this rule of thumb. Indeed, one of the main focuses of our study is that our Bayesian model estimates good prediction accuracy based on multi-locus/simultaneous association of all SNPs using small data set. This approach in predicting human disease phenotype using the Bayesian multi-locus association model is supported by closely related prediction models in plant breeding research. For example, using 80 markers and 126 soybean lines, Hu et al. demonstrated an increase from 33 to 78 % in explaining the phenotypic variance when markers were collectively accounted for epistatic effects [53]. The findings of Heffner et al. based on a wheat breeding program using a small set of samples [54] and markers also agree with our findings from human studies that jointly estimating all marker effects is able to capture more of the genetic variance than the two-stage conventional approach of first selecting significant markers from independent analysis and then estimating their effects.

Besides, phenotypic prediction using 100’s of SNPs, our Bayesian model provided the genetic effect estimated as WGV for each of the SNPs incorporated in the model and thus allowing to rank individual SNPs for exploring their functional role in the pathophysiology of the disease. For example, in our in-data prediction analysis, the top 110 SNPs with over 90 % accuracy were spread on 32 of 39 genes (25 CNS and 7 immune function candidate genes). It is interesting to note that SNPs from 7 out of 9 immune function genes were represented in this group that showed greater prediction, and SNPs in three of them (*IL12B* rs2288831, *IL1A* rs2071376, and *IFNG* rs2069718) showed the highest association with CFS in terms of WGV values. All three SNPs showed replicated or moderate association with other diseases as well. SNP rs2288831 is in complete LD with rs3212227 located in the 3′-untranslated region (3′UTR) of *IL12B* (Table 2), and this proxy SNP was reported to be associated with psoriasis in a large-scale association study, confirming the results of a previous study [55]. rs2071376 located in intron 6 of *IL1A* showed significant association in patients with keratoconus [56]. SNP rs2069718, located in the intron of *IFNG*, was associated with susceptibility to systemic lupus erythematosus in a recessive genetic model [57]. Among the CNS-related genes, the highest measures of genetic effects on CFS prediction were provided by SNPs in *HSD11B1* (rs846906), *HTR2A* (rs1923884), MAOB (rs1799836), *CRHR1* (rs1396862), *SLC18A2* (rs363236), *NOS3* (rs891512), and *DRD2* (rs1124492). Except rs1396862 and rs363236, these SNPs in CNS-related genes were located in introns with no potential function through transcription factor binding or splicing regulation or no proxy SNPs with functional significance. SNP rs891512, although located in intron of *NOS3*, showed protective effect against suicidal behavior [58]. While rs363236 is located in the 3′UTR of *SLC18A2* and is in complete LD with a marker (rs3814230) resulting in synonymous codon change in PDZD8, no functional role or association with disease could be identified with this marker. SNP rs1396862, on the other hand, is in complete LD with rs12185233 that results in a missense codon (R460P) change in *intramembrane protease 5 (IMP5*), a gene associated with Parkinson’s disease [59]. Another SNP in *CRHR1* (rs173365) with WGV of 0.58 also showed high LD with another missense codon change (rs242944, H302R) in *IMP5*, suggesting further support of association of this gene region with CFS. Genes with >5 SNPs that contributed to >90 % accuracy included *ACE*, *DRD2*, *HTR2A*, *HTR2C*, *HTR4*, *IL1A*, *MAOA*, *NR3C1*, and *TPH2*. Thus, at least 16 unique genes (*ACE*, *CRHR1*, *DRD2*, *HSD11B1*, *HTR2A*, *HTR2C*, *HTR4*, *IL12B*, *IL1A*, *IFNG*, *MAOA*, *MAOB*, *NOS3*, *NR3C1*, *SLC18A2*, and *TPH2)* appear to be major contributors to CFS prediction by greater genetic effects either through individual or multiple (>5) SNPs. Among these 16 genes, only two (*TPH2* and *NR3C1*) were common with the smaller set of 10 genes in the previous reports [17, 46] illustrating that higher predictive accuracy by multi-locus association is determined by variants with greater genetic effects. Sequence variations in some of these genes were reported to be associated with CFS (*NR3C1*: Rajeevan et al. [60] and *HTR2A*: Smith et al. [27]) or associated with some of the CFS subtypes identified by latent class analyses (*MAOA*, *MAOB*, *TPH2*, and *NR3C1*: Smith et al. [61]) or association with allostatic load, a construct that describes cumulative physiological effects of adaptation in response to stress (*ACE*: Smith et al. [62]). Besides estimating prediction accuracy of complex phenotype using multi-locus association, these findings on the association of individual markers/genes with CFS support the hypothesized general applicability of Bayesian model-based WGV estimates to identify specific genetic variations that may play biological roles in the pathophysiology of various diseases.

We concentrated more on the modeling principle rather than implementing the tool for routine phenotype prediction in this study. Thus, we used a general purpose software tool, WinBUGS, for MCMC estimation, which is often slower than tailor-made programs. Moreover, for routine phenotype prediction, maximum a posteriori estimation may be more practical than MCMC estimation (cf. [35, 63]). As is well known, population structure and cryptic relatedness are confounding factors in genetic association studies [64]. We have not corrected for these factors in our models since subjects were collected from a homogeneous population (Caucasians >93 % in both CFS and NF subjects) with no close relatives or complex links between individuals. However, even though such confounding factors may exist in the CFS data, the multi-locus association models have been found to be surprisingly robust to these confounders [31, 65]. Non-genetic risk factors can be included into the model as environmental covariates even if we have not done so here, and their inclusion may improve the accuracy even further. Presently, there is limited clinical/diagnostic utility for this genetic prediction model, since its reproducible performance remains to be evaluated in multiple populations. Further, in order to be clinically useful, it may be advantageous to evaluate prediction models by decision analytic techniques to determine whether models would change medical decisions. This Bayesian model provides an approach to improve phenotypic prediction by exploiting all available genetic information in 100’s of loci jointly, an approach that can be extended to the emerging computational field of whole-genome markers-enabled prediction of genetic predisposition in humans.

In conclusion, our results demonstrate, as a proof of principle, the power of using a combination of a Bayesian logistic mixture modeling principle, pathway-focused SNPs, and rigorous subject ascertainment for highly accurate prediction of complex phenotypes. WGV estimates provided by the model can also be useful to identify individual genetic markers/genes with potential biological functions. Future studies are warranted to expand this approach using multiple biological pathways and multiple populations [66] to derive a reproducible genetic profile with greater predictive power than non-genetic risk factors to identify chronic diseases like CFS and its subtypes that have no laboratory-based diagnosis or intermediate markers.

## Additional file

Additional file 1:
**Supplementary information. Figure S1.** Scatter plot showing the SNP level differences in the genetic effects estimated under the unconstrained and constrained (with the coefficient of class with higher frequency fixed at zero). **Figure S2.** Box plots of standard deviations over the 167 non-zero genetic effects, where standard deviations were calculated as follows: For each SNP and each (non-zero) genetic effect, the standard deviation over tenfolds was computed from the prior and also from the posterior distribution (under the constrained model used for tenfold cross-validation). **Table S1.** List of genes (along with functions) and SNPs tested for CFS prediction by the Bayesian logistic mixture model. Genes primarily belong to the central nervous system (CNS) including hypothalamic-pituitary-adrenal (HPA) pathway or immune function systems. **Table S2.** Weighted genetic variation in decreasing order for SNPs under full model.

## References

[1] de los Campos G, Gianola D, Allison DB (2010). Predicting genetic predisposition in humans: the promise of whole-genome markers. Nat Rev Genet.

[2] Jostins L, Barrett JC (2011). Genetic risk prediction in complex disease. Hum Mol Genet..

[3] Jakobsdottir J, Gorin MB, Conley YP, Ferrell RE, Weeks DE (2009). Interpretation of genetic association studies: markers with replicated highly significant odds ratios may be poor classifiers. PLoS Genet..

[4] Maher BS (2008). The case of missing heritability. Nature..

[5] Manolio TA, Collins FS, Cox NJ, Goldstein DB, Hindorff LA, Hunter DJ (2009). Finding the missing heritability of complex diseases. Nature..

[6] Lee SH, Wray NR, Goddard ME, Visscher PM (2011). Estimating missing heritability for disease from genome-wide association studies. Am J Hum Genet..

[7] Yang J, Benyamin B, McEvoy BP, Gordon S, Henders AK, Nyholt DR (2010). Common SNPs explain a large proportion of the heritability for human height. Nat Genet..

[8] Yang J, Lee SH, Goddard ME, Visscher PM (2011). GCTA: a tool for genome-wide complex trait analysis. Am J Hum Genet..

[9] O'Hara RB, Sillanpää MJ (2009). A review of Bayesian variable selection methods: what, how and which. Bayesian Anal..

[10] Zhou X, Carbonetto P, Stephens M (2013). Polygenic modeling with Bayesian sparse linear mixed models. PLoS Genet..

[11] Li Z, Sillanpää MJ (2012). Overview of LASSO-related penalized regression methods for quantitative trait mapping and genomic selection. Theor Appl Genet..

[12] Wu TT, Chen YF, Hastie T, Sobel E, Lange K (2009). Genome-wide association analysis by lasso penalized logistic regression. Bioinformatics..

[13] de los Campos G, Naya H, Gianola D, Crossa J, Legarra A, Manfredi E (2009). Predicting quantitative traits with regression models for dense molecular markers and pedigree. Genetics.

[14] de los Campos G, Hickey JM, Pong-Wong R, Daetwyler HD, Calus MP (2013). Whole-genome regression and prediction methods applied to plant and animal breeding. Genetics.

[15] Lee SH, van der Werf JH, Hayes BJ, Goddard ME, Visscher PM (2008). Predicting unobserved phenotypes for complex traits from whole-genome SNP data. PLoS Genet..

[16] de los Campos G, Vazquez AI, Fernando R, Klimentidis YC, Sorensen D (2013). Prediction of complex human traits using the genomic best linear unbiased predictor. PLoS Genet.

[17] Huang LC, Hsu SY, Lin E (2009). A comparison of classification methods for predicting chronic fatigue syndrome based on genetic data. J Transl Med..

[18] Bhattacharjee M, Sillanpää MJ. Bayesian joint disease-marker-expression analysis applied to clinical characteristics of chronic fatigue syndrome. In: McConnell P, Lin S, Cuticchia AJ, editors. Methods of microarray data analysis VI. (CAMDA). CAMDA 2009, 15–34.

[19] Bhattacharjee M, Botting CH, Sillanpää MJ (2008). Bayesian biomarker identification based on marker-expression proteomics data. Genomics..

[20] Bhattacharjee M, Sillanpää MJ (2011). A Bayesian mixed regression based prediction of quantitative traits from molecular marker and gene expression data. PLoS One..

[21] West M, Ginsburg GS, Huang AT, Nevins JR (2006). Embracing the complexity of genomic data for personalized medicine. Genome Res..

[22] Kabán A (2007). On Bayesian classification with Laplace priors. Patt Rec Lett..

[23] Sillanpää MJ, Bhattacharjee M (2005). Bayesian association-based fine mapping in small chromosomal segments. Genetics..

[24] Vernon SD, Reeves WC (2006). The challenge of integrating disparate high-content data: epidemiological, clinical and laboratory data collected during an in-hospital study of chronic fatigue syndrome. Pharmacogenomics..

[25] Reeves WC, Wagner D, Nisenbaum R, Jones JF, Gurbaxani B, Solomon L (2005). Chronic fatigue syndrome—a clinically empirical approach to its definition and study. BMC Med..

[26] Fukuda K, Straus SE, Hickie I, Sharpe MC, Dobbins JG, Komaroff A (1994). The chronic fatigue syndrome: a comprehensive approach to its definition and study. International Chronic Fatigue Syndrome Study Group. Ann Intern Med..

[27] Smith AK, Dimulescu I, Falkenberg VR, Narasimhan S, Heim C, Vernon SD (2008). Genetic evaluation of the serotonergic system in chronic fatigue syndrome. Psychoneuroendocrinology..

[28] Narita M, Nishigami N, Narita N, Yamaguti K, Okado N, Watanabe Y (2003). Association between serotonin transporter gene polymorphism and chronic fatigue syndrome. Biochem Biophys Res Commun..

[29] Saccone SF, Bolze R, Thomas P, Quan J, Mehta G, Deelman E (2010). SPOT: a web-based tool for using biological databases to prioritize SNPs after a genome-wide association study. Nucleic Acids Res..

[30] Fan J, Song R (2010). Sure independence screening in generalized linear models with NP-dimensionality. Ann Stat..

[31] Kärkkäinen HP, Sillanpää MJ (2012). Robustness of Bayesian multilocus association models to cryptic relatedness. Ann Hum Genet..

[32] Sasieni PD (1997). From genotypes to genes: doubling the sample size. Biometrics..

[33] Gao X, Haritunians T, Marjoram P, McKean-Cowdin R, Torres M, Taylor KD (2012). Genotype imputation for Latinos using the HapMap and 1000 genomes project reference panels. Front Genet..

[34] Servin B, Stephens M (2007). Imputation-based analysis of association studies: candidate regions and quantitative traits. PLoS Genet..

[35] Mutshinda CM, Sillanpää MJ (2012). Swift block-updating EM and pseudo-EM procedures for Bayesian shrinkage analysis of quantitative trait loci. Theor Appl Genet..

[36] Stone M (1974). Cross-validatory choice and assessment of statistical predictions. J Roy Stat Soc B..

[37] Hastie T, Tibshirani R, Friedman J. The elements of statistical learning: data mining, inference, and prediction. 2nd ed. Springer; 2009.

[38] Spiegelhalter DJ, Thomas A, Best NG (1999). WinBUGS Version 1.2 User Manual.

[39] Goeman JJ (2010). L-1 penalized estimation in the Cox proportional hazards model. Biometrical J..

[40] Conti DV, Witte JS (2003). Hierarchical modeling of linkage disequilibrium: genetic structure and spatial relations. Am J Hum Genet..

[41] Malo N, Libiger O, Schork NJ (2008). Accommodating linkage disequilibrium in genetic-association analyses via ridge regression. Am J Hum Genet..

[42] Tsai MY, Hsiao CK, Wen SH (2008). A Bayesian spatial multimarker genetic random-effect model for fine-scale mapping. Ann Hum Genet..

[43] Fridley BL, Jenkins GD (2010). Localizing putative markers in genetic association studies by incorporating linkage disequilibrium into Bayesian hierarchical models. Hum Hered..

[44] Yang W, Tempelman RJ (2012). A Bayesian antedependence model for whole genome prediction. Genetics..

[45] Yi H, Breheny P, Imam N, Liu Y, Hoeschele I (2015). Penalized multimarker vs. single-marker regression methods for genome-wide association studies of quantitative traits. Genetics..

[46] Goertzel BN, Pennachin C, de Souza CL, Gurbaxani B, Maloney EM, Jones JF (2006). Combinations of single nucleotide polymorphisms in neuroendocrine effector and receptor genes predict chronic fatigue syndrome. Pharmacogenomics..

[47] Zuber V, Duarte Silva AP, Strimmer K (2012). A novel algorithm for simultaneous SNP selection in high-dimensional genome-wide association studies. BMC Bioinf..

[48] Janssens AC, van Duijn CM (2008). Genome-based prediction of common diseases: advances and prospects. Hum Mol Genet..

[49] Loza MJ, McCall CE, Li L, Isaacs WB, Xu J, Chang BL (2007). Assembly of inflammation-related genes for pathway-focused genetic analysis. PLoS One..

[50] Hattori E, Liu C, Zhu H, Gershon ES (2005). Genetic tests of biologic systems in affective disorders. Mol Psychiatry..

[51] Sillanpää MJ, Corander J (2002). Model choice in gene mapping: what and why. Trends Genet..

[52] Meuwissen TH (2009). Accuracy of breeding values of ‘unrelated’ individuals predicted by dense SNP genotyping. Genet Sel Evol..

[53] Hu Z, Li Y, Song X, Han Y, Cai X, Xu S (2011). Genomic value prediction for quantitative traits under the epistatic model. BMC Genet..

[54] Heffner EL, Jannink J-L, Sorrells ME (2011). Genome selection accuracy using multifamily prediction models in a wheat breeding program. Plant Genome..

[55] Cargill M, Schrodi SJ, Chang M, Garcia VE, Brandon R, Callis KP (2007). A large-scale genetic association study confirms IL12B and leads to the identification of IL23R as psoriasis-risk genes. Am J Hum Genet..

[56] Kim SH, Mok JW, Kim HS, Joo CK (2008). Association of −31T > C and −511 C > T polymorphisms in the interleukin 1 beta (IL1B) promoter in Korean keratoconus patients. Mol Vis..

[57] Kim K, Cho SK, Sestak A, Namjou B, Kang C, Bae SC (2010). Interferon-gamma gene polymorphisms associated with susceptibility to systemic lupus erythematosus. Ann Rheum Dis..

[58] Rujescu D, Giegling I, Mandelli L, Schneider B, Hartmann AM, Schnabel A (2008). NOS-I and -III gene variants are differentially associated with facets of suicidal behavior and aggression-related traits. Am J Med Genet B Neuropsychiatr Genet..

[59] Edwards TL, Scott WK, Almonte C, Burt A, Powell EH, Beecham GW (2010). Genome-wide association study confirms SNPs in SNCA and the MAPT region as common risk factors for Parkinson disease. Ann Hum Genet..

[60] Rajeevan MS, Smith AK, Dimulescu I, Unger ER, Vernon SD, Heim C (2007). Glucocorticoid receptor polymorphisms and haplotypes associated with chronic fatigue syndrome. Genes Brain Behav..

[61] Smith AK, White PD, Aslakson E, Vollmer-Conna U, Rajeevan MS (2006). Polymorphisms in genes regulating the HPA axis associated with empirically delineated classes of unexplained chronic fatigue. Pharmacogenomics..

[62] Smith AK, Maloney EM, Falkenberg VR, Dimulescu I, Rajeevan MS (2009). An angiotensin-1 converting enzyme polymorphism is associated with allostatic load mediated by C-reactive protein, interleukin-6 and cortisol. Psychoneuroendocrinology..

[63] Li Z, Sillanpää MJ (2012). Estimation of quantitative trait locus effects with epistasis by variational Bayes algorithms. Genetics..

[64] Sillanpää MJ (2010). Overview of techniques to account for confounding due to population stratification and cryptic relatedness in genomic data association analyses. Heredity..

[65] Pikkuhookana P, Sillanpää MJ (2009). Correcting for relatedness in Bayesian models for genomic data association analysis. Heredity..

[66] Sillanpää MJ, Bhattacharjee M (2006). Association mapping of complex trait loci with context-dependent effects and unknown context variable. Genetics..

