# Supplementary material for: Prediction of complex human diseases from pathway-focused candidate markers by joint estimation of marker effects: case of chronic fatigue syndrome
Source: Hum Genomics. 2015 Jun 11;9(1):8. doi: 10.1186/s40246-015-0030-6 (PMC4479222; doi:10.1186/s40246-015-0030-6)
Supplement: Additional file 1: — Supplementary information. Figure S1. Scatter plot showing the SNP level differences in the genetic effects estimated under the unconstrained and constrained (with the coefficient of class with higher frequency fixed at zero). Figure S2. Box plots of standard deviations over the 167 non-zero genetic effects, where standard deviations were calculated as follows: For each SNP and each (non-zero) genetic effect, the standard deviation over tenfolds was computed from the prior and also from the posterior distribution (under the constrained model used for tenfold cross-validation). Table S1. List of genes (along with functions) and SNPs tested for CFS prediction by the Bayesian logistic mixture model. Genes primarily belong to the central nervous system (CNS) including hypothalamic-pituitary-adrenal (HPA) pathway or immune function systems. Table S2. Weighted genetic variation in decreasing order for SNPs under full model. [file 40246_2015_30_MOESM1_ESM.doc]

**SUPPLEMENTARY MATERIALS**

**Prediction of Complex Human Diseases from Pathway-Focused Candidate Markers by Joint Estimation of Marker Effects: Case of Chronic Fatigue Syndrome**

Madhuchhanda Bhattacharjee,^1^ Mangalathu S. Rajeevan,^2^ and Mikko J. Sillanpää^3,4^

^1^ School of Mathematics and Statistics, University of Hyderabad, Hyderabad-500046, India

^2^ Division of High-Consequence Pathogens and Pathology, Centers for Disease Control and Prevention, Atlanta, USA 30333

^3^ Departments of Mathematics and Statistics, and Department of Agricultural Sciences, University of Helsinki, FIN-00014 Finland

^4^ Departments of Mathematical Sciences and Biology, Biocenter Oulu, University of Oulu, FIN-90014 Finland

**Weighted genetic variation and heuristic model reduction**

The weighted genetic variation (WGV_l_=|*β_l,1_ - β_l,2_*|*I_l_* ) at locus *l*, (*l = 1, …, M*), is computed as a product of absolute difference of the genetic effects |*β_l,1_ - β_l,2_*| and the inclusion indicator (*I_l_*). WGV is used as a summary statistic for each location. It is defined as a model averaged posterior estimate of genetic variance calculated as the product of the indicator variable times the variance (or absolute difference) of genetic effects of the SNP. We would like to clarify that our usage of the term “WGV” is not meant to capture the QTL-heritability or the amount of genetic variance explained by the genetic markers in the population-level. With this term we simply refer to individual-level genomic predicted value calculated over markers and variation is occurring only over different models which are weighted.

A heuristic model reduction method was used to speed up the estimation by determining the number of SNP predictors that can be reduced without loss of predictive ability. Using the percentiles of WGV (Supplementary Table 2) as cutoff/threshold, a selected set of SNPs was retained in the model with two critical components derived from the overall model as follows. For the indicators, the joint posterior distribution of the indicators from the full model was used where the outcome of the spike-n-slab technique over the MCMC simulation for full model was stored and re-used for model reduction purposes.

For the remaining SNPs (with WGV lower than threshold value) the individual level genotype information were not used and thus these SNPs would effectively cease to act as covariates in the model. The overall model would still need to be adjusted for these SNPs since we prefer not to re-simulate without these as covariates in the model. Thus, instead of using individual-level genotype information, population-level information could be used. Here population is defined as that used for original parameter estimation which are proportions of two homozygotes and heterozygote in the CFS-NF (Supplementary Table 2). This procedure leads to revision of the intercept term using the original estimate of intercept based on full model, the parameters related to the removed SNPs and their population level allelic frequency estimates.

Assume that the full model has *M* SNPs and we have posterior samples from *2M+1* parameters (i.e. *2M* parameters for the genetic effects and *1* for the intercept). In the revised notation the effect coefficients include both genetic effects and inclusion indicators. Therefore $\beta_{i,l}^{1}= (\beta_{l,1}(2-m_{i,l})+\beta_{l,2}m_{i,l}) I_{l}$ , *I_l_* : Indicator for SNP-*l,* $m_{i,l}$: The genotype value of subject $i$at SNP-*l*, $\beta_{l,k}$: *k*-th genetic effect of SNP *l*, *k=1,2*, *l=1, …, M*, *i= 1, …, n* and *M_S_*: SNPs on Sex-chromosomes, *M_A_*: SNPs on autosomal chromosomes. So the logistic model for the *i*-th subject will appear to be

$$Logit(p_{i}) = \alpha+ \sum_{l\in M_{A}} \beta_{i,l}^{1} + \frac{1}{2}\sum_{l\in M_{S}} \beta_{i,l}^{1}$$

For prediction we would use a set of samples from the posterior of these parameters and for the *j*-th MCMC sample the prediction for the *i*-th individual is carried out using the following regression formula:

$Logit(p_{ij}) = \alpha_{j} + \sum_{l\in M_{A}} \beta_{i,l,j}^{1} + \frac{1}{2}\sum_{l\in M_{S}} \beta_{i,l,j}^{1}$.

As mentioned earlier, now we reduce the model to M_1_ SNPs and use population proportions for the remaining M-M_1_ SNPs. In this exercise we would still be using the same posterior sample for the selected M_1_ SNPs. However the adjustment we are making for the omitted SNPs using population proportion would result in a revised intercept term (which is common to all subjects). Thus in effect we are using a revised sample for the intercept term α.

Our approximate prediction is done using the following:

$Logit(p_{ij}) = \alpha_{j}^{r} + \sum_{l\in M_{A1}} \beta_{i,l,j}^{1} + \frac{1}{2}\sum_{l\in M_{S1}} \beta_{i,l,j}^{1}$,

Where M_S1_: selected SNPs on Sex-chromosomes, M_A1_: selected SNPs on autosomal chromosomes (with M_1_ = |M_S1_| +|M_A1_|and

$$\begin{matrix} \alpha_{j}^{r} = \alpha_{j} + \sum_{M_{A1}^{c}} I_{l} \left(2\beta_{l,1}f_{11} + 2\beta_{l,2}f_{22} + \left(\beta_{l,1}+\beta_{l,2}\right)f_{12}\right) \\ + \frac{1}{2}\sum_{M_{S1}^{c}} I_{j} \left(2\beta_{l,1}f_{11} + 2\beta_{l,2}f_{22} + \left(\beta_{l,1}+\beta_{l,2}\right)f_{12}\right), \end{matrix}$$

Here *f_11_*: frequency of homozygotes type *1, f_22_*: frequency of homozygotes type *2, f_12_*: frequency of heterozygotes, M^c^_A1_: complement of M_A1_ in M_A_, that is SNPs omitted from M_A_, M^c^_S1_: complement of M_S1_ in M_S_, that is SNPs omitted from M_S_.

In our approximate prediction based on the smaller model, we revised the intercept term using the original intercept term, the allelic coefficients and population frequencies for omitted SNPs. The MCMC sample thus revised presents a sample from the 2M_1_+1 dimensional parameter space of interest.

***K*-fold cross-validation**

Cross validation methods [36] give a better assessment of model predictive performance for new data, i.e. phenotypic predictions of individuals whose phenotypes and genotypes have not been involved in the learning sample. The hold-out or split-sample method, in which the data is split into training and testing sets, is the simplest kind of cross validation. While this method assesses model performance on real prediction situation with new data it is subjective to the choice of the partition of the data into training and testing sets. *K*-fold cross validation is one way to improve over the split-sample method. The data set is divided into *K* (approximately) equal subsets, and the holdout method is repeated *K* times, by which every data point gets to be in a test set exactly once, and gets to be in a training set *K*-1 times. We have used *K*=10 which is also one of the most popular choices of *K* [37].

One major disadvantage of *K*-fold cross validation is that the time taken would also be typically *K* times that required for estimation based on whole data. Using data on all 167 SNPs, a 10-fold cross validation would require approximately 150 minutes per MCMC iteration. We retained information from all SNPs since CFS phenotype is complex and there could be loss of predictive ability with reduced number of SNPs. Therefore, instead of reducing the number of SNPs in the model to reduce the time, we made a few modifications to the full model (Section 2.3) as noted below.

As discussed in Section 2.3 while introducing the model for and(i.e. the genetic effects) a common practice is to constrain orto zero and estimate the other one as free parameter. We slightly modified our proposed model in Section 2.3 in the following manner: the prior distribution for genetic effects *β_l_,*_1_ and *β_l_,*_2_ were still assumed to be normal with locus-specific variance component where the value for the coefficient of the class with higher frequency is held fixed at zero. Thus the joint prior of andwould still be a *2M* dimensional distribution as in the proposed model; however, half of them would be evaluated at the prefixed value of 0. Our experience suggests that this method can substantially reduce computational time per iteration of MCMC sampling. The (posterior) estimates obtained under this constrained model are comparable to those obtained without the constraints (i.e. model described in Section 2.3). Supplementary Figure 1 presents the SNP level differences in the genetic effects estimated under the unconstrained and constrained models (with the coefficient of class with higher frequency fixed at zero). However, models with direct constraint on parameters can (and in this case does) cause difficulties in finding suitable updated values for the parameters during MCMC computation, resulting in possibly longer simulation. This is one of the reasons to employ the unconstrained model proposed in Section 2.3.

In our setup, the prediction rule refers to the application of the posterior distribution of the parameters concerned on the logistic model described earlier. Effectively we are trying to assess how robust this distribution is when we account for sampling variation. In our case this variation is captured by the 10-folds of the original data set created. To assess the robustness we center the distributions of some of the key parameters (but not all) on their respective posterior means as obtained by the model in Section 2.3. However we allow a relatively uninformative prior around it. The parameters modeled in this method are the intercept term (*α)* and the (non-zero) genetic effects (*β).*

Thus, where and is the posterior mean of the intercept as obtained by implementing the proposed model on the whole data. Similar assumptions were made for the distributions of the genetic effects described as follows, and thus with and is the posterior mean of the coefficient. This is implemented with the additional constraints on the values of the coefficients for the frequent class for the SNPs.

First, it was confirmed that the whole data estimation of parameters was comparable to the unconstrained model described in Section 2.3. Once this was verified, a model for 10-fold cross validation was implemented. The prior variation over the 10-folds around the “posterior” means allowed under this model is found to be substantially higher than posterior variation over the 10-folds (See Supplementary Figure 2). Supplementary Figure 2 shows box plots of standard deviations over the 167 non-zero genetic effects, where standard deviations have been calculated as follows. For each SNP and each (non-zero) genetic effect the standard deviation over the 10-folds were computed from the prior and also from the posterior distribution (under the constrained model used for 10-fold cross validation). Thus this model in-effect implements a broad prior around the means estimated using the whole data to check their robustness over sampling variability (as captured by the 10-folds).

**Supplementary Figure 1**: Scatter plot showing the SNP level differences in the genetic effects estimated under the unconstrained and constrained (with the coefficient of class with higher frequency fixed at zero).

|  |
| --- |
| **Supplementary Figure 2**. Box plots of standard deviations over the 167 non-zero genetic effects, where standard deviations were calculated as follows: For each SNP and each (non-zero) genetic effect the standard deviation over the 10-folds were computed from the prior and also from the posterior distribution (under the constrained model used for 10-fold cross validation). |
|  |
|  |

**Supplementary Table 1: List of genes (along with functions) and SNPs tested for CFS prediction by the Bayesian logistic mixture model. Genes primarily belong to the central nervous system (CNS) including hypothalamic- pituitary-adrenal pathway (HPA) or Immune function systems.**

| Chromosome | Gene symbol | Gene name | Function Group | Function | SNPs (NCBI rsID) | Number of SNPs tested |
| --- | --- | --- | --- | --- | --- | --- |
| 1 | *HTR6* | *5-Hydroxytryptamine (serotonin) receptor 6* | CNS | Serotonin receptor activity | rs1805054, rs6684942 | 2 |
| 1 | *IL10* | *Interleukin 10* | Immune | Cytokine with effects on immune and inflammation | rs1554286, rs1518111 | 2 |
| 1 | *HSD11B1* | *Hydroxysteroid (11-beta)  dehydrogenase 1* | CNS | Convert cortisol to cortisone | rs2884090, rs846906 | 2 |
| 2 | *POMC* | *Proopiomelanocortin* | CNS | Polypeptide precursor of ACTH | rs12473543 | 1 |
| 2 | *IL1A* | *Interleukin 1, alpha* | Immune | Cytokine activity, synthesized in response to cell injury | rs1304037, rs2071376, rs2071375, rs17561, rs2071373, rs1894399 | 6 |
| 2 | *IL1B* | *Interleukin 1, beta* | Immune | Cytokine activity on cell proliferation, differentiation and apoptosis | rs1143643, rs1143634, rs1143629 | 3 |
| 2 | *HTR2B* | *5-Hydroxytryptamine (serotonin) receptor 2B* | CNS | Serotonin receptor activity | rs765458 | 1 |
| 3 | *DRD3* | *Dopamine receptor D3* | CNS | Dopamine receptor | rs3773678 | 1 |
| 4 | *SPP1* | *Secreted phosphoprotein 1* | Immune | Differential regulation of IL12 and IL10 | rs11730582, rs1126616, rs1126772 | 3 |
| 5 | *SLC6A3* | *Solute carrier family 6, member 3* | CNS | Dopamine transporter | rs10064219, rs40184 | 2 |
| 5 | *HTR1A* | *5-Hydroxytryptamine  (serotonin) receptor 1A* | CNS | Serotonin receptor activity | rs878567, rs6295 | 2 |
| 5 | *NR3C1* | *Nuclear receptor subfamily 3, group C, member 1* | CNS | Receptor for glucocorticoids | rs6198, rs6191, rs6196, rs258750, rs6188, rs852977, rs860458, rs2918419, rs1866388 | 9 |
| 5 | *HTR4* | *5-Hydroxytryptamine (serotonin) receptor 4* | CNS | Serotonin receptor activity | rs6889822, rs10037493, rs7733410, rs7716435, rs1883074, rs13159589, rs9325104, rs2895768, rs35558914, rs980062, rs13166230, rs10477387, rs4289549, 3168086 | 14 |
| 5 | *IL12B* | *Interleukin 12B* | Immune | Cytokine with effects on T and NK cells | rs1368439, rs2853696, rs2288831 | 3 |
| 6 | *HTR1E* | *5-Hydroxytryptamine (serotonin) receptor 1E* | CNS | Serotonin receptor activity | rs10944288 | 1 |
| 7 | *IL6* | *Interleukin 6* | Immune | Proinflammatory cytokine | rs1800795, rs2069845 | 2 |
| 7 | *CRHR2* | *Corticotropin releasing hormone receptor 2* | CNS | CNS | rs2284217 | 1 |
| 7 | *NOS3* | *Nitric oxide synthase 3* | CNS | CNS | rs1800779, rs1007311, rs1800780, rs891512 | 4 |
| 7 | *HTR5A* | *5-Hydroxytryptamine (serotonin) receptor 5A* | CNS | CNS | rs1800883, rs1079515, rs731107, rs732050 | 4 |
| 8 | *INDO* | *Indoleamine 2,3-dioxygenase 1* | CNS and immune | CNS and immune | rs10108662, rs3739319 | 2 |
| 9 | *DBH* | *Dopamine beta-hydroxylase* | CNS | CNS | rs1611125, rs739398, rs2073833, rs2073837 | 4 |
| 10 | *HTR7* | *5-Hydroxytryptamine (serotonin) receptor 7* | CNS | CNS | rs12412496, rs12413941, rs12766851, rs2185706 | 4 |
| 10 | *SLC18A2* | *Solute carrier family 18, member 2* | CNS | CNS | rs363390, rs363343, rs929493, rs363236 | 4 |
| 11 | *TH* | *Tyrosine hydroxylase* | CNS | CNS | rs2070762, rs4074905 | 2 |
| 11 | *BDNF* | *Brain-derived  neurotrophic factor* | CNS | CNS | rs6265, rs11030104, rs2049045, rs7103411 | 4 |
| 11 | *DRD2* | *Dopamine*  *receptor D2* | CNS | CNS | rs1124492, rs1124493, rs1079595, rs1107162, rs2075654, rs2002453, rs2245805, rs1125393, rs12800853, rs11214608, rs7117915 | 11 |
| 11 | *HTR3B* | *5-Hydroxytryptamine (serotonin) receptor 3B* | CNS | CNS | rs1176744, rs2276307, rs2276308, rs11607692 | 4 |
| 11 | *HTR3A* | *5-hydroxytryptamine (serotonin) receptor 3A* | CNS | CNS | rs1150222, rs1176719, rs1150220, rs1176713 | 4 |
| 12 | *TNFRSF1A* | *Tumor necrosis factor receptor superfamily, member 1A* | Immune | Immune | rs1860545, 2645708, 2645705 | 3 |
| 12 | *IFNG* | *Interferon, gamma* | Immune | Immune | rs2069718 | 1 |
| 12 | *TPH2* | *Tryptophan hydroxylase 2* | CNS | CNS | rs10784941, rs2171363, rs4760816, rs4760750, rs1386486, rs1487280, rs1872824 | 7 |
| 13 | *HTR2A* | *5-Hydroxytryptamine (serotonin) receptor 2A* | CNS | CNS | rs6314, rs1923882, rs1923884, rs1923885, rs655888, rs643627, rs6313, rs6311, rs9534512 | 9 |
| 17 | *SLC6A4* | *Solute carrier family 6, member 4* | CNS | CNS | rs2066713 | 1 |
| 17 | *CRHR1* | *Corticotropin releasing hormone receptor 1* | CNS | CNS | rs7209436, rs110402, rs242924, rs242940, rs173365, rs1396862 | 6 |
| 17 | *ACE* | *Angiotensin I-converting enzyme* | Immune | Immune | rs4295, rs4978, rs4575595, rs11868324, rs4461142, rs4267385, rs4968591 | 7 |
| 22 | *COMT* | *Catechol-O-methyltransferase* | CNS | CNS | rs933271, rs5993882, rs740603, rs4646312, rs165722, rs6269, rs4633 | 7 |
| X | *MAOA* | *Monoamine oxidase A* | CNS | CNS | rs5906893, rs909525, rs1800464, rs6323, rs979606, rs979605, rs1137070 | 7 |
| X | *MAOB* | *Monoamine oxidase A* | CNS | CNS | rs1799836, rs2283729 | 2 |
| X | *HTR2C* | *5-Hydroxytryptamine (serotonin) receptor 2C* | CNS | CNS | rs505971, rs12558586, rs1023574, rs2069237, rs2497543, rs6318, rs2497535, rs2257137, rs2497523, rs2497514, rs7066090, rs5946194, rs6643915, rs1335614 | 14 |

**Supplementary Table 2. Weighted genetic variation in decreasing order for SNPs under full model**

| Sr. no. | Gene symbol | NCBI rsID | Chr. | Position | Homo-zygous-1 | Homo-zygous-2 | Hetero-zygous | Weighted genetic variation |
| --- | --- | --- | --- | --- | --- | --- | --- | --- |
| 51 | *IL12B* | rs2288831 | 5 | 158682591 | 0.10 | 0.65 | 0.25 | 3.95 |
| 9 | *IL1A* | rs2071376 | 2 | 113251866 | 0.07 | 0.60 | 0.33 | 3.60 |
| 106 | *IFNG* | rs2069718 | 12 | 66836429 | 0.12 | 0.36 | 0.52 | 3.34 |
| 6 | *HSD11B1* | rs846906 | 1 | 207954341 | 0.75 | 0.02 | 0.23 | 3.29 |
| 116 | *HTR2A* | rs1923884 | 13 | 46319837 | 0.48 | 0.07 | 0.46 | 3.16 |
| 152 | *MAOB* | rs1799836 | X | 43512943 | 0.25 | 0.32 | 0.42 | 2.56 |
| 129 | *CRHR1* | rs1396862 | 17 | 41258778 | 0.03 | 0.57 | 0.40 | 2.31 |
| 77 | *SLC18A2* | rs363236 | 10 | 119028361 | 0.02 | 0.81 | 0.17 | 2.31 |
| 59 | *NOS3* | rs891512 | 7 | 150339022 | 0.04 | 0.70 | 0.26 | 2.18 |
| 84 | *DRD2* | rs1124492 | 11 | 112787485 | 0.05 | 0.77 | 0.18 | 2.02 |
| 78 | *TH* | rs2070762 | 11 | 2142911 | 0.26 | 0.28 | 0.46 | 1.54 |
| 48 | *HTR4* | rs4289549 | 5 | 148002363 | 0.36 | 0.16 | 0.49 | 1.40 |
| 49 | *IL12B* | rs1368439 | 5 | 158674592 | 0.03 | 0.65 | 0.32 | 1.39 |
| 74 | *SLC18A2* | rs363390 | 10 | 118994069 | 0.09 | 0.59 | 0.32 | 1.37 |
| 37 | *HTR4* | rs7733410 | 5 | 147836715 | 0.15 | 0.41 | 0.45 | 1.36 |
| 131 | *ACE* | rs4978 | 17 | 58927493 | 0.33 | 0.29 | 0.39 | 1.35 |
| 117 | *HTR2A* | rs1923885 | 13 | 46321087 | 0.17 | 0.34 | 0.50 | 1.33 |
| 155 | *HTR2C* | rs12558586 | X | 113751903 | 0.76 | 0.03 | 0.21 | 1.29 |
| 17 | *HTR2B* | rs765458 | 2 | 231698911 | 0.11 | 0.43 | 0.47 | 1.24 |
| 36 | *HTR4* | rs10037493 | 5 | 147835163 | 0.39 | 0.15 | 0.47 | 1.23 |
| 103 | *TNFRSF1A* | rs1860545 | 12 | 6317038 | 0.13 | 0.41 | 0.46 | 1.22 |
| 136 | *ACE* | rs4968591 | 17 | 58951850 | 0.13 | 0.37 | 0.50 | 1.21 |
| 133 | *ACE* | rs11868324 | 17 | 58931041 | 0.19 | 0.29 | 0.52 | 1.19 |
| 45 | *HTR4* | rs980062 | 5 | 147947692 | 0.40 | 0.11 | 0.50 | 1.10 |
| 76 | *SLC18A2* | rs929493 | 10 | 119009116 | 0.05 | 0.65 | 0.30 | 1.09 |
| 111 | *TPH2* | rs1386486 | 12 | 70698487 | 0.14 | 0.41 | 0.46 | 1.07 |
| 154 | *HTR2C* | rs505971 | X | 113721064 | 0.49 | 0.17 | 0.35 | 1.07 |
| 108 | *TPH2* | rs2171363 | 12 | 70646531 | 0.17 | 0.41 | 0.42 | 1.05 |
| 19 | *SPP1* | rs11730582 | 4 | 89115445 | 0.26 | 0.22 | 0.52 | 1.04 |
| 1 | *HTR6* | rs1805054 | 1 | 19865100 | 0.80 | 0.02 | 0.18 | 1.03 |
| 57 | *NOS3* | rs1007311 | 7 | 150326941 | 0.33 | 0.24 | 0.44 | 1.02 |
| 18 | *DRD3* | rs3773678 | 3 | 115352768 | 0.02 | 0.75 | 0.23 | 1.01 |
| 42 | *HTR4* | rs2895768 | 5 | 147906430 | 0.40 | 0.12 | 0.49 | 1.01 |
| 34 | *NR3C1* | rs1866388 | 5 | 142739978 | 0.42 | 0.11 | 0.47 | 0.96 |
| 8 | *IL1A* | rs1304037 | 2 | 113248707 | 0.07 | 0.48 | 0.44 | 0.94 |
| 10 | *IL1A* | rs2071375 | 2 | 113251909 | 0.48 | 0.07 | 0.45 | 0.91 |
| 97 | *HTR3B* | rs2276308 | 11 | 113309186 | 0.58 | 0.01 | 0.41 | 0.91 |
| 21 | *SPP1* | rs1126772 | 4 | 89123210 | 0.56 | 0.06 | 0.38 | 0.87 |
| 157 | *HTR2C* | rs2069237 | X | 113825403 | 0.02 | 0.78 | 0.20 | 0.86 |
| 40 | *HTR4* | rs13159589 | 5 | 147902255 | 0.40 | 0.12 | 0.48 | 0.85 |
| 32 | *NR3C1* | rs860458 | 5 | 142676229 | 0.08 | 0.70 | 0.22 | 0.83 |
| 162 | *HTR2C* | rs2497523 | X | 113903282 | 0.01 | 0.80 | 0.19 | 0.83 |
| 119 | *HTR2A* | rs643627 | 13 | 46326612 | 0.05 | 0.53 | 0.42 | 0.81 |
| 153 | *MAOB* | rs2283729 | X | 43562986 | 0.07 | 0.53 | 0.40 | 0.81 |
| 85 | *DRD2* | rs1124493 | 11 | 112787505 | 0.45 | 0.16 | 0.40 | 0.80 |
| 50 | *IL12B* | rs2853696 | 5 | 158677238 | 0.66 | 0.04 | 0.30 | 0.80 |
| 13 | *IL1A* | rs1894399 | 2 | 113256648 | 0.48 | 0.06 | 0.47 | 0.80 |
| 72 | *HTR7* | rs12766851 | 10 | 92552811 | 0.54 | 0.06 | 0.40 | 0.80 |
| 120 | *HTR2A* | rs6313 | 13 | 46367941 | 0.38 | 0.17 | 0.45 | 0.79 |
| 163 | *HTR2C* | rs2497514 | X | 113912932 | 0.80 | 0.01 | 0.19 | 0.79 |
| 166 | *HTR2C* | rs6643915 | X | 114042839 | 0.81 | 0.01 | 0.18 | 0.79 |
| 159 | *HTR2C* | rs6318 | X | 113871991 | 0.02 | 0.78 | 0.20 | 0.79 |
| 114 | *HTR2A* | rs6314 | 13 | 46307035 | 0.87 | 0.02 | 0.11 | 0.79 |
| 165 | *HTR2C* | rs5946194 | X | 113983781 | 0.01 | 0.80 | 0.19 | 0.77 |
| 35 | *HTR4* | rs6889822 | 5 | 147826900 | 0.49 | 0.10 | 0.42 | 0.76 |
| 43 | *HTR4* | None | 5 | 147911890 | 0.48 | 0.13 | 0.40 | 0.75 |
| 160 | *HTR2C* | rs2497535 | X | 113883118 | 0.76 | 0.02 | 0.22 | 0.75 |
| 29 | *NR3C1* | rs258750 | 5 | 142642082 | 0.40 | 0.15 | 0.45 | 0.74 |
| 158 | *HTR2C* | rs2497543 | X | 113870478 | 0.02 | 0.78 | 0.20 | 0.74 |
| 98 | *HTR3B* | rs11607692 | 11 | 113323993 | 0.00 | 0.82 | 0.18 | 0.74 |
| 11 | *IL1A* | rs17561 | 2 | 113253694 | 0.07 | 0.48 | 0.45 | 0.73 |
| 92 | *DRD2* | rs12800853 | 11 | 112807724 | 0.21 | 0.33 | 0.46 | 0.73 |
| 148 | *MAOA* | rs6323 | X | 43475980 | 0.20 | 0.52 | 0.28 | 0.72 |
| 161 | *HTR2C* | rs2257137 | X | 113893410 | 0.77 | 0.01 | 0.22 | 0.72 |
| 38 | *HTR4* | rs7716435 | 5 | 147853590 | 0.16 | 0.34 | 0.50 | 0.72 |
| 147 | *MAOA* | rs1800464 | X | 43456141 | 0.91 | 0.00 | 0.09 | 0.71 |
| 151 | *MAOA* | rs1137070 | X | 43488335 | 0.50 | 0.19 | 0.31 | 0.70 |
| 30 | *NR3C1* | rs6188 | 5 | 142660537 | 0.15 | 0.40 | 0.45 | 0.70 |
| 12 | *IL1A* | rs2071373 | 2 | 113256555 | 0.50 | 0.07 | 0.44 | 0.70 |
| 150 | *MAOA* | rs979605 | X | 43486307 | 0.19 | 0.51 | 0.30 | 0.70 |
| 113 | *TPH2* | rs1872824 | 12 | 70716581 | 0.18 | 0.42 | 0.41 | 0.70 |
| 164 | *HTR2C* | rs7066090 | X | 113956346 | 0.01 | 0.81 | 0.18 | 0.70 |
| 112 | *TPH2* | rs1487280 | 12 | 70705094 | 0.16 | 0.40 | 0.45 | 0.70 |
| 33 | *NR3C1* | rs2918419 | 5 | 142702546 | 0.08 | 0.70 | 0.22 | 0.69 |
| 167 | *HTR2C* | rs1335614 | X | 114053710 | 0.79 | 0.01 | 0.20 | 0.69 |
| 156 | *HTR2C* | rs1023574 | X | 113761276 | 0.17 | 0.49 | 0.35 | 0.68 |
| 145 | *MAOA* | rs5906893 | X | 43412568 | 0.50 | 0.19 | 0.31 | 0.65 |
| 82 | *BDNF* | rs2049045 | 11 | 27650817 | 0.01 | 0.77 | 0.22 | 0.65 |
| 96 | *HTR3B* | rs2276307 | 11 | 113309097 | 0.56 | 0.01 | 0.43 | 0.65 |
| 132 | *ACE* | rs4575595 | 17 | 58930947 | 0.32 | 0.27 | 0.42 | 0.64 |
| 63 | *HTR5A* | rs732050 | 7 | 154504328 | 0.50 | 0.10 | 0.40 | 0.64 |
| 26 | *NR3C1* | rs6198 | 5 | 142637814 | 0.65 | 0.04 | 0.31 | 0.64 |
| 89 | *DRD2* | rs2002453 | 11 | 112794508 | 0.54 | 0.10 | 0.36 | 0.63 |
| 94 | *DRD2* | rs7117915 | 11 | 112838635 | 0.01 | 0.71 | 0.28 | 0.63 |
| 149 | *MAOA* | rs979606 | X | 43486086 | 0.20 | 0.51 | 0.29 | 0.63 |
| 115 | *HTR2A* | rs1923882 | 13 | 46309662 | 0.61 | 0.09 | 0.30 | 0.63 |
| 71 | *HTR7* | rs12413941 | 10 | 92536386 | 0.00 | 0.54 | 0.46 | 0.61 |
| 93 | *DRD2* | rs11214608 | 11 | 112820565 | 0.25 | 0.31 | 0.45 | 0.60 |
| 70 | *HTR7* | rs12412496 | 10 | 92536145 | 0.00 | 0.56 | 0.44 | 0.59 |
| 7 | *POMC* | rs12473543 | 2 | 25240685 | 0.02 | 0.66 | 0.32 | 0.59 |
| 75 | *SLC18A2* | rs363343 | 10 | 119004938 | 0.61 | 0.08 | 0.31 | 0.59 |
| 118 | *HTR2A* | rs655888 | 13 | 46326182 | 0.06 | 0.53 | 0.41 | 0.58 |
| 128 | *CRHR1* | rs173365 | 17 | 41256855 | 0.16 | 0.30 | 0.54 | 0.58 |
| 138 | *COMT* | rs933271 | 22 | 18311407 | 0.04 | 0.51 | 0.45 | 0.58 |
| 31 | *NR3C1* | rs852977 | 5 | 142667687 | 0.40 | 0.16 | 0.45 | 0.58 |
| 121 | *HTR2A* | rs6311 | 13 | 46369479 | 0.38 | 0.17 | 0.45 | 0.57 |
| 24 | *HTR1A* | rs878567 | 5 | 63291747 | 0.25 | 0.22 | 0.53 | 0.56 |
| 73 | *HTR7* | rs2185706 | 10 | 92593125 | 0.00 | 0.76 | 0.24 | 0.56 |
| 90 | *DRD2* | rs2245805 | 11 | 112795909 | 0.55 | 0.10 | 0.35 | 0.55 |
| 146 | *MAOA* | rs909525 | X | 43438146 | 0.22 | 0.48 | 0.31 | 0.55 |
| 100 | *HTR3A* | rs1176719 | 11 | 113357397 | 0.02 | 0.64 | 0.34 | 0.54 |
| 15 | *IL1B* | rs1143634 | 2 | 113306861 | 0.07 | 0.61 | 0.32 | 0.53 |
| 55 | *CRHR2* | rs2284217 | 7 | 30680133 | 0.02 | 0.61 | 0.37 | 0.53 |
| 109 | *TPH2* | rs4760816 | 12 | 70658868 | 0.16 | 0.40 | 0.45 | 0.52 |
| 126 | *CRHR1* | rs242924 | 17 | 41241147 | 0.30 | 0.16 | 0.54 | 0.52 |
| 28 | *NR3C1* | rs6196 | 5 | 142641683 | 0.72 | 0.05 | 0.23 | 0.52 |
| 62 | *HTR5A* | rs731107 | 7 | 154503736 | 0.45 | 0.12 | 0.44 | 0.52 |
| 86 | *DRD2* | rs1079595 | 11 | 112787879 | 0.69 | 0.02 | 0.29 | 0.52 |
| 135 | *ACE* | rs4267385 | 17 | 58937488 | 0.18 | 0.33 | 0.50 | 0.51 |
| 83 | *BDNF* | rs7103411 | 11 | 27656701 | 0.04 | 0.74 | 0.22 | 0.51 |
| 87 | *DRD2* | rs1107162 | 11 | 112794247 | 0.34 | 0.25 | 0.42 | 0.49 |
| 39 | *HTR4* | rs1883074 | 5 | 147853943 | 0.17 | 0.35 | 0.49 | 0.49 |
| 81 | *BDNF* | rs11030104 | 11 | 27641093 | 0.75 | 0.02 | 0.23 | 0.49 |
| 41 | *HTR4* | rs9325104 | 5 | 147903570 | 0.32 | 0.19 | 0.50 | 0.49 |
| 2 | *HTR6* | rs6684942 | 1 | 19871588 | 0.03 | 0.16 | 0.81 | 0.48 |
| 142 | *COMT* | rs165722 | 22 | 18329013 | 0.22 | 0.30 | 0.49 | 0.48 |
| 25 | *HTR1A* | rs6295 | 5 | 63294321 | 0.19 | 0.21 | 0.60 | 0.48 |
| 110 | *TPH2* | rs4760750 | 12 | 70664156 | 0.16 | 0.40 | 0.45 | 0.47 |
| 137 | *SLC6A4* | None | 17 |  | 0.35 | 0.17 | 0.48 | 0.47 |
| 23 | *SLC6A3* | rs40184 | 5 | 1448077 | 0.22 | 0.30 | 0.49 | 0.46 |
| 124 | *CRHR1* | rs7209436 | 17 | 41225913 | 0.28 | 0.15 | 0.57 | 0.45 |
| 20 | *SPP1* | rs1126616 | 4 | 89122877 | 0.47 | 0.10 | 0.42 | 0.45 |
| 3 | *IL10* | rs1554286 | 1 | 205010856 | 0.05 | 0.71 | 0.24 | 0.45 |
| 14 | *IL1B* | rs1143643 | 2 | 113304773 | 0.40 | 0.10 | 0.50 | 0.45 |
| 105 | *TNFRSF1A* | None | 12 | 6319376 | 0.42 | 0.17 | 0.42 | 0.45 |
| 80 | *BDNF* | rs6265 | 11 | 27636492 | 0.76 | 0.01 | 0.23 | 0.45 |
| 61 | *HTR5A* | rs1079515 | 7 | 154497192 | 0.45 | 0.13 | 0.43 | 0.45 |
| 144 | *COMT* | rs4633 | 22 | 18330235 | 0.24 | 0.30 | 0.47 | 0.44 |
| 4 | *IL10* | rs1518111 | 1 | 205011268 | 0.68 | 0.05 | 0.27 | 0.44 |
| 127 | *CRHR1* | rs242940 | 17 | 41248380 | 0.23 | 0.18 | 0.58 | 0.43 |
| 47 | *HTR4* | rs10477387 | 5 | 147993935 | 0.09 | 0.44 | 0.48 | 0.43 |
| 44 | *HTR4* | rs35558914 | 5 | 147922597 | 0.43 | 0.13 | 0.45 | 0.43 |
| 125 | *CRHR1* | rs110402 | 17 | 41235818 | 0.15 | 0.28 | 0.57 | 0.43 |
| 68 | *DBH* | rs2073833 | 9 | 135510103 | 0.26 | 0.23 | 0.51 | 0.42 |
| 46 | *HTR4* | rs13166230 | 5 | 147978569 | 0.48 | 0.10 | 0.43 | 0.42 |
| 99 | *HTR3A* | rs1150222 | 11 | 113352082 | 0.80 | 0.02 | 0.18 | 0.42 |
| 79 | *TH* | rs4074905 | 11 | 2145761 | 0.54 | 0.10 | 0.36 | 0.42 |
| 134 | *ACE* | rs4461142 | 17 | 58931780 | 0.34 | 0.18 | 0.49 | 0.42 |
| 140 | *COMT* | rs740603 | 22 | 18325177 | 0.20 | 0.24 | 0.56 | 0.42 |
| 104 | *TNFRSF1A* | None | 12 | 6317783 | 0.26 | 0.27 | 0.48 | 0.42 |
| 22 | *SLC6A3* | rs10064219 | 5 | 1437998 | 0.01 | 0.77 | 0.22 | 0.41 |
| 91 | *DRD2* | rs1125393 | 11 | 112802559 | 0.69 | 0.02 | 0.29 | 0.41 |
| 141 | *COMT* | rs4646312 | 22 | 18328337 | 0.15 | 0.35 | 0.49 | 0.41 |
| 143 | *COMT* | rs6269 | 22 | 18329952 | 0.36 | 0.16 | 0.49 | 0.40 |
| 139 | *COMT* | rs5993882 | 22 | 18317533 | 0.00 | 0.56 | 0.44 | 0.40 |
| 101 | *HTR3A* | rs1150220 | 11 | 113363096 | 0.02 | 0.69 | 0.29 | 0.39 |
| 95 | *HTR3B* | rs1176744 | 11 | 113308238 | 0.46 | 0.04 | 0.50 | 0.39 |
| 102 | *HTR3A* | rs1176713 | 11 | 113365635 | 0.64 | 0.02 | 0.34 | 0.38 |
| 54 | *IL6* | rs2069845 | 7 | 22736674 | 0.30 | 0.26 | 0.45 | 0.37 |
| 16 | *IL1B* | rs1143629 | 2 | 113309989 | 0.39 | 0.09 | 0.52 | 0.37 |
| 65 | *INDO* | rs3739319 | 8 | 39904478 | 0.17 | 0.37 | 0.47 | 0.36 |
| 69 | *DBH* | rs2073837 | 9 | 135512749 | 0.11 | 0.36 | 0.53 | 0.35 |
| 130 | *ACE* | rs4295 | 17 | 58910030 | 0.19 | 0.49 | 0.33 | 0.35 |
| 52 | *HTR1E* | rs10944288 | 6 | 87708461 | 0.08 | 0.51 | 0.41 | 0.34 |
| 66 | *DBH* | rs1611125 | 9 | 135499133 | 0.29 | 0.19 | 0.52 | 0.34 |
| 107 | *TPH2* | rs10784941 | 12 | 70622779 | 0.24 | 0.27 | 0.50 | 0.34 |
| 122 | *HTR2A* | rs9534512 | 13 | 46371922 | 0.25 | 0.34 | 0.42 | 0.34 |
| 88 | *DRD2* | rs2075654 | 11 | 112794276 | 0.69 | 0.02 | 0.29 | 0.33 |
| 53 | *IL6* | rs1800795 | 7 | 22733170 | 0.32 | 0.22 | 0.46 | 0.32 |
| 58 | *NOS3* | rs1800780 | 7 | 150329812 | 0.16 | 0.32 | 0.52 | 0.32 |
| 67 | *DBH* | rs739398 | 9 | 135506391 | 0.29 | 0.32 | 0.40 | 0.31 |
| 60 | *HTR5A* | rs1800883 | 7 | 154493524 | 0.14 | 0.52 | 0.34 | 0.31 |
| 5 | *HSD11B1* | rs2884090 | 1 | 207950682 | 0.63 | 0.08 | 0.29 | 0.30 |
| 56 | *NOS3* | rs1800779 | 7 | 150320876 | 0.40 | 0.14 | 0.46 | 0.30 |
| 123 | *SLC6A4* | rs2066713 | 17 | 25575791 | 0.18 | 0.36 | 0.47 | 0.29 |
| 27 | *NR3C1* | rs6191 | 5 | 142638349 | 0.23 | 0.27 | 0.49 | 0.27 |
| 64 | *INDO* | rs10108662 | 8 | 39899146 | 0.12 | 0.44 | 0.45 | 0.26 |

**WinBUGS code (with 10-fold cross validation, i.e. kf=10):**

model {

# Modelling genotype

for( j in 1 : m1 ) {

for( k in 1 : n2 ) {

p1[ j, k ] <- 0.5

}

for( i in 1 : n1 ) {

for( k in 1 : 2 ) {

y[ k, i, j ] ~ dcat( p1[ j, 1:n2 ] )

}

}

}

# Model for the indicators

lambda ~dgamma(1, 0.01)

for( j in 1 : m1-1 ) {

q[ j ] <- exp( (-1)*lambda*d[ j ] )

w[ j ] ~ dbern( q[ j ] )

}

for( l in 1 : kf ) {

x[ l, 1 ] ~ dbern( s )

for( j in 2 : m1 ) {

x0[ l, j ] ~ dbern( s )

x[l,j]<-w[j-1]*x[l,j-1]+(1-w[j-1])*x0[l,j]

}

x1[ l ] <- sum( x[ l, 1:m1 ] )

for( j in 1 : m1+1 ) {

x2[ l, j ] <- equals( x1[ l ] , j-1 )

}

}

# Modelling phenotype variable

tau0 ~ dgamma(1, 1)

for( j in 1 : m1 ) {

for( k in 1 : n2 ) {

tau1[ j, k ] ~dgamma(1, 1)

}

}

for( l in 1 : kf ) {

# mu[ l ] ~ dnorm( 0 , tau0 )I(-1000, 1000)

mu[ l ] ~ dnorm( mu0, tau0 )I(-1000, 1000)

for( j in 1 : m1 ) {

for( k in 1 : n2 ) {

# beta[l,j,k] ~ dnorm( 0 ,tau1[j,k])I(-1000, 1000)

beta[l,j,k] ~ dnorm(beta0[j,k],tau1[j,k])I(-1000,1000)

}

}

for( i in 1 : n1) {

for( j in 1 : m1 ) {

beta1[l,i,j]<-x[l,j]*(beta[l,j,y[1,i,j]]+beta[l,j,y[2,i,j]])

}

logit(s1[l,i])<-mu[l]+sum(beta1[l,i,1:m11]) +(0.5*sum(beta1[l,i,m12:m13]))

z1[ l, i ] ~ dbern( s1[ l, i ] )

}

}

# Predicting phenotype

for( i in 1 : n1) {

zz1[ 1, i ] <- s1[ set[ i ], i ]

zz1[ 2, i ] <- ( sum( s1[ 1:kf, i ] ) - zz1[ 1, i ] ) / (kf-1)

zpred[ i ] <- step( zz1[ 1, i ] - 0.5 )

}

# Prediction assessment

tp <- sum( zpred[ 59 : 101 ])

fn <- ( 43 - tp )

fp <- sum( zpred[ 1 : 58 ] )

tn <- ( 58 - fp )

tmp1 <- tp + tn

zall[ 1 ] <- (100 * tp) / ( tp + fn ) # sn

zall[ 2 ] <- (100 * tn) / ( tn + fp ) # sp

zall[ 3 ] <- (100 * fp) / ( tp + fp ) # fdr

zall[ 4 ] <- ( tmp1* 100) / n1 # accuracy

zall[ 5 ] <- (100 * tp) / ( tp + fp ) # ppv

zall[ 6 ] <- ((tp*tn)-(fp*fn))/(sqrt((tp+fp)*(tp+fn)*(tn+fp)*(tn+fn))) # cc

zall[ 7 ] <- fp / tp # tpc

}

# **************** Input data ********************************

list(

n1= 101, # Number of subjects

m1=167, # Number of markers

m11=144, # Number of autosomal markers

m12=145, m13=167, # Other markers

n2 = 2, # Number of alleles (vector for non-SNP type markers)

d = c( ), # Between marker distances

s = 0.5, # Apriori probability (independently of LD)

# of a marker being associated to phenotype

kf = 10, # Number of folds for cross validation

set = c( ), # Fold membership of a subject

mu0 = -0.205, # Hyper-parameters, typically set to zero

beta0 = structure(.Data = c( ), .Dim = c( 167, 2 )),

# Hyper-parameters, typically set to zero

z1 = # Phenotype data for learning sets

structure(.Data = c(

0, 0, 0, 0, 0, 0, 0, 0, 0, 0, 0, 0, 0, 0, 0, 0, 0, 0, 0, 0, 0, 0, 0, 0, 0, 0, 0, NA, 0, 0, 0, 0, 0, 0, 0, 0, 0, 0, NA, 0, NA, 0, 0, 0, NA, NA, 0, NA, 0, 0, 0, 0, 0, 0, 0, 0, 0, 0, 1, 1, NA, 1, 1, 1, NA, NA, 1, 1, 1, 1, NA, 1, 1, 1, 1, 1, 1, 1, 1, 1, 1, 1, 1, 1, 1, 1, 1, 1, 1, 1, 1, 1, 1, 1, 1, NA, 1, 1, 1, 1, 1,

………………..

0, 0, 0, NA, 0, 0, 0, 0, NA, 0, 0, 0, 0, 0, 0, 0, 0, 0, 0, 0, 0, 0, 0, 0, 0, 0, 0, 0, 0, NA, 0, 0, 0, 0, 0, 0, 0, 0, 0, 0, 0, 0, 0, 0, 0, 0, NA, 0, 0, NA, 0, 0, 0, 0, 0, 0, 0, 0, NA, 1, 1, 1, 1, 1, 1, 1, 1, NA, 1, 1, 1, 1, 1, 1, 1, 1, 1, 1, 1, 1, 1, NA, 1, 1, 1, NA, 1, 1, NA, 1, 1, 1, 1, 1, 1, 1, 1, 1, 1, 1, 1 ), .Dim = c( 10, 101 )),

y = # Genotype data

structure(.Data = c(

1,1,2,1,1,1,2,1,2,2,1,1,2,1,1,1,2,2,1,1,1,2,2,1,2,1,1,1,1,2,1,2,2,1,1,1,2,2,2,1,1,1,1,1,1,1,2,1,2,1,2,1,1,1,1,1,1,1,1,2,2,1,1,1,2,2,2,1,2,1,1,2,1,1,2,1,2,1,1,1,1,2,2,2,1,1,1,1,1,1,1,1,1,2,1,1,1,2,1,2,2,1,2,2,1,1,2,2,2,2,2,2,2,NA,2,2,2,1,1,1,1,1,1,2,1,2,2,2,2,1,1,2,1,1,1,1,2,1,NA,2,2,2,1,1,1,2,1,2,2,2,1,2,2,1,1,1,1,1,1,1,1,1,1,1,1,1,1,

…………………………………….

1,2,2,1,1,1,1,2,2,1,2,2,1,1,2,2,2,2,1,2,2,2,2,1,1,1,1,1,1,2,1,2,2,1,1,2,2,2,2,NA,1,1,1,1,1,1,2,1,2,2,2,2,2,2,2,2,2,2,2,2,1,1,1,2,1,1,2,2,1,2,2,1,2,2,1,2,2,2,1,2,2,2,1,2,1,1,1,1,1,1,1,2,2,2,2,2,2,2,1,2,2,1,2,2,1,2,2,2,2,2,2,2,2,1,2,2,2,2,2,2,2,2,2,2,1,2,2,2,2,1,2,2,2,2,2,2,2,2,2,1,1,1,1,2,1,1,1,2,2,2,1,1,2,2,2,2,2,2,2,2,2,2,2,2,2,2,2

), .Dim = c( 2, 101, 167 ))

)
